# Shared Species Analysis, Augmented by Stochasticity Analysis, Is More Effective Than Diversity Analysis in Detecting Variations in the Gut Microbiomes

**DOI:** 10.3389/fmicb.2022.914429

**Published:** 2022-07-19

**Authors:** Zhanshan (Sam) Ma

**Affiliations:** ^1^Computational Biology and Medical Ecology Lab, Kunming Institute of Zoology, Chinese Academy of Sciences, Kunming, China; ^2^Center for Excellence in Animal Genetics and Evolution, Chinese Academy of Sciences, Kunming, China

**Keywords:** Chinese gut microbiome (CGM), shared species analyses (SSA), Hill numbers (diversity), permutation tests, rural vs. urban lifestyle, ethnicity, stochasticity analysis

## Abstract

Diversity analysis is a *de facto* standard procedure for most existing microbiome studies. Nevertheless, diversity metrics can be insensitive to changes in community composition (identities). For example, if species A (*e.g*., a beneficial microbe) is replaced by equal number of species B (*e.g*., an opportunistic pathogen), the diversity metric may not change, but the community composition has changed. The shared species analysis (SSA) is a computational technique that can discern changes of community composition by detecting the increase/decrease of shared species between two sets of microbiome samples, and it should be more sensitive than standard diversity analysis in discerning changes in microbiome structures. Here, we investigated the effects of ethnicity and lifestyles in China on the structure of Chinese gut microbiomes by reanalyzing the datasets of a large Chinese cohort with 300+ individuals covering 7 biggest Chinese ethnic groups (>95% Chinese population). We found: (*i*) Regarding lifestyles, SSA revealed significant differences between 100% of pair-wise comparisons in community compositions across all but phylum taxon levels (phylum level = 29%), but diversity analysis only revealed 14–29% pair-wise differences in community diversity across all four taxon levels. (*ii*) Regarding ethnicities, SSA revealed 100% pair-wise differences in community compositions across all but phylum (phylum level = 48–62%) levels, but diversity analysis only revealed 5–57% differences in community diversity across all four taxon levels. (*iii*) Ethnicity seems to have more prevalent effects on community structures than lifestyle does (*iv*) Community structures of the gut microbiomes are more stable at the phylum level than at the other three levels. (*v*) SSA is more powerful than diversity analysis in detecting the changes of community structures; furthermore, SSA can produce lists of unique and shared OTUs. (*vi*) Finally, we performed *stochasticity* analysis to mechanistically interpret the observed differences revealed by the SSA and diversity analyses.

## Introduction

The human gut microbiomeand its host constitute an ecosystem of symbiotic, commensal, and pathogenic microorganisms that are 10+ times more than the number of our somatic cells. In the co-evolutionary process of intestinal microbes and hosts, both intrinsic host factors and external environmental factors jointly influence the human intestinal micro-ecosystem and gut microbiomes that directly and/or indirectly affect our health and diseases. Among the numerous factors that influence the human gut microbiome, lifestyles(*e.g.*, the rural *vs*. urban) and ethnic identities are arguably two of the most influential (Zhang et al., 2015; Deschasaux et al., 2018; Lin et al., 2020). Lifestyle is closely related to diet, ethnicity is closely related to host genetic background, and both are obviously among the most variables that determine the structure and dynamics of our gut microbiomes.

Measuring the changes of gut microbiome structure is critical to microbiome research. Diversity analysis has been one of the most commonly performed techniques for investigating microbiome structure and dynamics. Indeed, diversity metrics play a foundational role in community ecology, and play an indispensable role for biodiversity research and conservation practice. Using an analogy, the foundational role of diversity metrics in community ecology is not unlike the role of statistical moments (such as mean, variance, skewness) in mathematical statistics. For example, two of the most important diversity metrics, Shannon entropy (Shannon, 1948) and Simpson index (Simpson, 1949), have been routinely computed in biodiversity studies of animals and plants since 1950's and, more recently, in the studies of human and other environmental microbiomes. Nevertheless, diversity metrics are imperfect in characterizing community structures because species identities are implicitly ignored and diversity is often defined as some kind of entropy function of species abundances, which hides species identity information implicitly. For instance, if species *A* is replaced by equal number of species *B*, the *diversity* metric may not change but the community *composition* has changed. If both species A and B have the same or similar functions, then the issue is less relevant. However, if both have different functions (*e.g*., A—a beneficial microbe; B—an opportunistic pathogen), then the constancy in diversity metric can be misleading. In this case, the shared species analysis (SSA) (Ma et al., 2019), a pair of computational algorithms (A1 and A2) for determining the changes of community (species) compositions, can play an important Supplementary Role. The SSA test does not simply compare the OTU richness or diversity of communities but, instead, quantifies the difference in species composition (OTU identity) between the two communities, which is similar to a measure of beta diversity in terms of Anderson et al. (2011). To strengthen the rigor of the SSA, Ma et al. (2019) proposed two algorithms (A1 and A2): A1 randomizes the assignments of the individual reads (bacterial individuals) to the rural or urban groups (for example), and A2 randomizes the assignments of the entire sample from a single subject (and its associated reads) to the rural or urban groups. A fundamental difference between both the algorithms is that A1 treats the individual reads as independent elements, whereas the more conservative A2 treats the entire sample of reads from a single subject as the independent sampling element.

In the present study, we apply SSA to investigate the effects of lifestyles and ethnicities on the human gut microbiome and further compare the SSA results with the results from standard diversity analysis. We choose to reanalyze a large Chinese gut microbiome dataset collected from 300+ individuals from 7 ethnic groups residing in typical country and urban settings. As China occupies a vast land and widely varying climates and environments, people in different regions have developed various dietary cultures (Zhang et al., 2015). Furthermore, the 56 ethnic Chinese groups constitute nearly 20% of the world population. The 300+ individuals were sampled from the top 7 biggest ethnic groups in China, including Han, Mongolians, Uyghurs, and Tibetans, Kazakh, Zhuang, and Bai, covering 95% of Chinese in China. For each of the 7 ethnic groups, individuals living rural and urban lifestyles were sampled separately. Each ethnic group except Han Chinese lives in a different geographical location, and its own dietary habits and particular lifestyles are preserved, especially Mongolians, Uyghurs, and Tibetans (Dehingia et al., 2015; Zhang et al., 2015). For example, rural Mongolians maintain a traditional nomadic lifestyle and have dietary tradition of high consumption of red meat, cheese, and liquor (Ley et al., 2006). The study showed that *Bifidobacterium spp*., *Enterobacter spp*., and *Enterococcus spp*. had high diversity and were the dominant bacteria in the intestinal tract of the Bai people in Dali, Yunnan Province, which may be related to the long-term consumption of flavored milk food made by a traditional and unique process by the local people (Huang et al., 2015). According to relevant studies, at the phylum level and the genus level, the keymicroorganisms that differ among Mongolians, Han Chinese, and Europeans are *Actinobacterium* and *Bifidobacterium*, respectively (Liu et al., 2016). Thus, ethnic origin should play an important role in the composition of human gut microbiome. Zhang et al. (2015) used principal component analysis to analyze the composition of the gut microbiome between individuals, which revealed significant differences between Mongolians and Tibetans, Mongolians and Zhuang, and Tibetans in rural and urban areas, and huge differences in the composition of intestinal microbiome between rural Tibetans and other ethnic groups (Zhang et al., 2015). In spite of extensive studies on the effects of diets, lifestyles and ethnicities, no simple metrics or statistical tests similar to diversity metrics (analysis) have been applied to approach the problem. In the present study, we filled the gap by applying the shared species analysis to the investigation of the lifestyle and ethnicity effects on the structural variations of Chinese gut microbiome. In particular, we aimed to demonstrate the power of shared species analysis in discerning microbiome compositional changes beyond what standard diversity analysis could deliver.

Beyond performing comparative SSA and diversity analyses of the Chinese gut microbiomes, a secondary objective of this study is to shed theoretic light on the *mechanistic* insights on the possible differences revealed by the SSA and diversity analyses. To pursue this objective, we resort to the ecological theory of metacommunity, which sought to expose the mechanisms underlying the assembly of ecological communities and the maintenance of their biodiversities in the context of metacommunity that consists of multiple local communities. Arguably, the first theory aimed to explain the community assembly, and diversity maintenance is the *niche* theory, which stipulates that natural habitats (such as the human gut) for living things (such as the gut microbes) are differentiated into niches suitable for different species to survive and prosper. The niche theory maintains that it is the deterministic selection forces that “drive” the species to their suitable niches and, therefore, shape the process of community assembly (formation) and patterns of community diversity (including composition).

According to niche theory, the dynamics of microbiome diversity and/or the succession of microbiome development (*e.g*., from infants to adults) should be deterministic, and are determined (or selected) by the interactions between microbial species and their niche environments (Grinnell, 1917; Vandermeer, 1972; Chase and Leibold, 2003). However, niche theory can hardly explain many non-deterministic phenomena in community ecology. In the late 1990's, an alternative theory to the niche theory, known as the unified neutral theory of biodiversity and biogeography (UNTB), was developed by Stephen Hubbell (Hubbell, 2001; Rosindell et al., 2011). Hubbell's UNTB took a virtually opposite view with niche theory, and it stipulates that stochastic drifts of species demography (birth, death, and migration) shape the patterns of biodiversity and biogeography. The UNTB maintains that biological species are neutral in the sense that, at a given trophic level, in a food web (community), species are equivalent in vital (birth/death/dispersal/speciation) rates on a *per capita* basis. According to the UNTB, stochastic drifts, rather than niche differentiations, are responsible for the observed species abundance distributions in ecological communities. In fact, the UNTB also takes into account the speciation and dispersal (migration) (Hubbell, 2001) besides stochastic drifts in vital rates, and, combined together, the theory forms the basis for testing, and frequently rejecting the null hypothesis (model), *i.e*., that communities are structured by demographic stochasticity alone (Rosindell et al., 2011).

While niche theory and neutral theory are two major and, also, diametrically opposing hypotheses about community assembly and diversity maintenance, the debates between two camps helped to advance more comprehensive theories in community ecology, most notably metacommunity theory (Leibold et al., 2004) and more recent four-process (mechanisms) synthesis of community ecology and biogeography (Vellend, 2010; Hanson et al., 2012). Vellend (2010) synthesis of community ecology maintains that selection, drift, speciation, and dispersal are the four key processes (mechanisms) that drive the community structure and dynamics, and Hanson et al. (2012) further extended the synthesis to the community dynamics and biogeography of microbes. Note that Hubbell's UNTB covers three of the four aspects of Vellend–Hanson synthesis (excluding *selection*).

Those further advances show that there is middle ground between niche theory and neutral theory, which means that both deterministic niche differentiations (selection forces) and stochastic neutral drifts can be in effects in assembling communities and maintaining their biodiversities (Hammal et al., 2015; Li and Ma, 2019; Ma, 2020a,b, 2021a,b). On the one hand, natural communities are often structured by stabilizing niche differences and competitive asymmetries among species, which typically generate distinctly non-neutral communities (Gilbert and Levine, 2017); both theoretic and experimental studies have demonstrated that stochastic neutral effects cannot be excluded in many natural communities. Several hybrid models that consider both niche differentiations and stochastic drifts have been developed to describe the middle ground quantitatively (Jeraldo et al., 2012; Harris et al., 2017). Indeed, there is so-termed niche-neutral continuum to conceptualize the continuous spectrum with one end of total deterministic niche selections and the other end of total stochastic neutral drifts. For many years, the concept of niche-neutral continuum has been treated as an analogy because of its lack of quantitative model. Nonetheless, a recent advance by Ning et al. (2019) seemed to have filled the gap; they developed a so-termed stochasticity analysis framework. Specifically, their normalized stochasticity ratio (NSR) with value ranging between *0* and *1*, represents the spectrum from total deterministic niche differentiations to stochastic neutral drifts.

In summary, the objective of this study is two-fold. We first performed comparative studies with the SSA (Ma et al., 2019) and diversity metrics (in Hill numbers) (Chao et al., 2012, 2014) to investigate the effects of ethnicities and lifestyles on the gut microbiome composition and diversity. Second, we applied stochasticity analysis (Ning et al., 2019) to seek mechanistic insights into the effects of ethnicity and/or lifestyles, and we postulate that those effects revealed by SSA/diversity metrics should be deterministic (non-stochastic). Although we use the datasets of Chinese gut microbiomes, our integrated approach of shared species, diversity, and stochasticity should be of general applicability to the microbiomes and macrobiomes in other environments.

## Materials and Methods

### Gut Microbiome Datasets and Study Design

**Figure 1:**
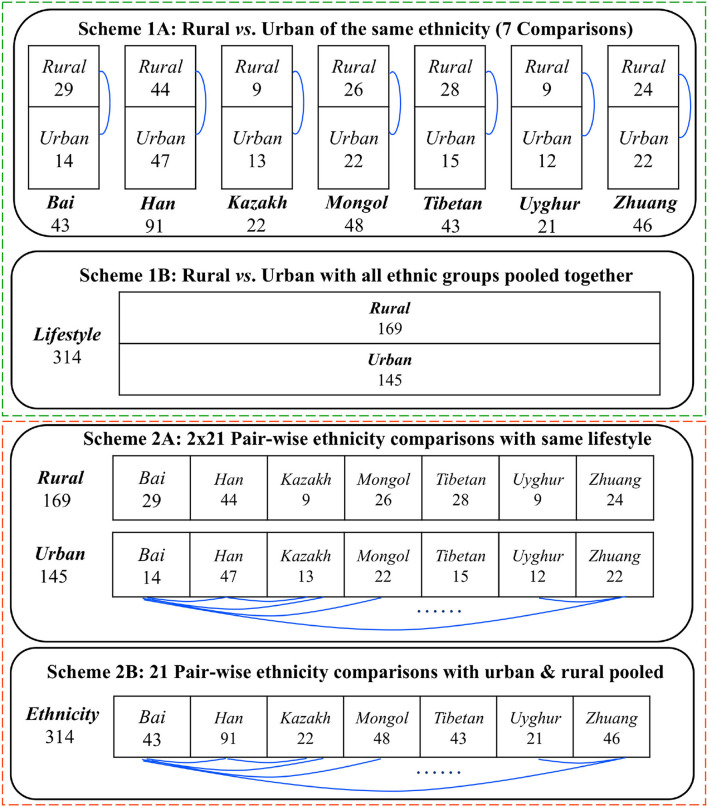
Four design schemes (1A−2B) for comparing the Chinese gut microbiomesfrom 7 major Chinese ethnic groups living in rural and urban settings, respectively.

Figure 1 illustrates our design schemes for reanalyzing the Chinese gut microbiome (CGM) datasets, originally collected by Zhang et al. (2015) that sampled 314 healthy Chinese adults, covering 7 largest Chinese ethnic races (groups) resided in rural and urban settings, respectively. All of the datasets reanalyzed in this study have already been published in Zhang et al. (2015), and, therefore, no experimental procedures are involved in the present article (no ethnic approval is applicable).

The top section (Schemes 1A,B) exhibits the design to test the effects of lifestyles (rural vs. urban) on the community structures of CGM. The difference between Schemes 1A,B lies in the treatment of potential confounding effects of ethnicity. In Scheme 1A, the analyses are performed for each of the 7 ethnic groups to test community structural changes between both the lifestyles within the focal ethnic group. A total of 7 comparisons (rural vs. urban lifestyles) are possible for Scheme 1A as illustrated in the first block of Figure 1. In Scheme 1B, the analyses are performed by pooling together the samples from the 7 ethnic groups under each lifestyle category and forming two “big” samples to test the community structural changes between the rural and urban lifestyles. With Scheme 1B, only one comparison is needed to compare the rural vs. urban lifestyles as illustrated in the second block of Figure 1. Obviously, the Scheme 1B can potentially contain confounding effects of ethnicity when testing the effects of lifestyles, and, therefore, we only use it as a supplement to Scheme 1A, and we should not draw independent conclusions from Scheme 1B.

The bottom section (Schemes 2A,B) displays the design to test the effects of ethnicity (7 ethnic groups in total) on the community structure of CGM. The difference between 2A and 2B lies in the treatment of potential confounding effects of lifestyles. In Scheme 2A, the analyses are performed for each of the two lifestyles to test pair-wise community structural changes between any two groups within the focal lifestyle. A total of 2-x-21 (42) comparisons [2^*^Combination (7, 2)] are possible for Scheme 2A as illustrated in the third block of Figure 1. In Scheme 2B, the analyses are performed by pooling together the samples from both the lifestyles under each ethnic group and forming seven “big” samples to test the community structural changes between pair-wise ethnic groups. With Scheme 2B, 21 comparisons [Combination (7, 2)] are needed as illustrated in the fourth block of Figure 1. Obviously, the Scheme 2B can potentially contain confounding effects of lifestyles when testing the effects of ethnicities, and, therefore, we only use it as a supplement to Scheme 2A, and we should not draw independent conclusions from Scheme 2B.

For each of the four design schemes, we performed both shared species analysis (SSA) based on two algorithms (A1 and A2) developed by Ma et al. (2019) and diversity analysis based on the Hill numbers (Chao et al., 2014; Ma and Li, 2018), which are briefly introduced below. Detailed information on both approaches is referred to their original methodology publications and beyond the scope of this paper. The focus of this article is to apply them, in comparative and integrated manner, for examining the effects of lifestyles and ethnicities on the community structures of Chinese gut microbiomes. Here, we narrowed community “structures” to community composition and diversity. The difference between the both is that, in the former, the taxonomic identities of species or OTUs (operational taxonomic units) are considered explicitly, while, in the latter, species identified are ignored, and only their abundances are considered. Obviously, ignoring species identities (*e.g*., beneficial microbes *vs*. opportunistic pathogens) may have far-reaching implications in microbiome research. This study is aimed to demonstrate that SSA should possess an indispensable role in microbiome studies beyond standard diversity analysis that has been routinely performed in current microbiome research.

### Shared Species (OTUs) Analysis

The term “shared species” or more general “shared OTUs” (operational taxonomic units) refer to those species (OTUs) with non-zero abundances in both treatments of microbiome samples. The number of shared species (or OTUs) between two cohorts (populations), *e.g*., with different lifestyles in rural vs. urban settings, may vary among studies, and depends in part on the number of individuals (microbiome samples) per cohort and the number of reads per individual sample. If there are distinctive OTUs associated with specific lifestyle, then there should be relatively few shared OTUs between two cohorts (populations) with different lifestyles. Alternatively, if the same microbiome is associated with rural and urban individuals, the distinctive OTUs in each group would represent random sampling effects (which are especially strong for rare or under-sampled taxa), and the number of shared OTUs would be no different than expected by chance (*H*_0_). The SSA compares the composition, similar to beta diversity (Anderson et al., 2011), of the treatments, whereas the following diversity analysis with Hill numbers compares the alpha diversity, or taxon richness, between treatments (lifestyles in this case).

Ma et al. (2019) developed two SSA algorithms (A1 and A2) to estimate the number of shared OTUs expected under *H*_0_. In A1 termed “reads randomization,” the expected number of shared OTUs was generated by pooling all the reads (bacterial individuals) within each pair-wise comparison (*e.g.*, the rural *vs*. urban lifestyles) together and then randomly assigning each read to the rural or urban category of samples. A1 does not change the total number of reads in each of the two original groups. In A2 termed “samples randomization,” we randomly assigned each microbiome sample in the study (pair-wise comparison) to the rural or urban group, and then pooled the reads within each of the randomized pseudo-groups. A2 does not change the numbers of microbiome samples in each of the two original groups.

After randomization with A1 or A2, the reads within each pseudo-group are pooled together, and the number of shared OTUs between the two pseudo-groups is computed. The randomization (permutation) is to repeat 1,000 times in order to generate a distribution of the expected number of shared OTUs under the null hypothesis of random sampling (*H*_0_). The observed number of OTUs is compared to the simulated distribution to estimate the tail probability of obtaining the observed results with random sampling *p*(*#Shared OTUs*|*H*_0_). The null-model results are further converted into a standardized effect size:


(1) $$\begin{array}{l} SES=\left[SOTU_{obs}-mean\left(SOTU_{sim}\right)\right]/sd\left(SOTU_{sim}\right) \end{array}$$

where *SOTU*_*obs*_ = the observed number of shared OTUs, *mean* (*SOTU*_*sim*_) = the average number of shared OTUs in the 1,000 simulated assemblages, and *sd* (*SOTU*_*sim*_) is the sample standard deviation of the 1,000 simulated assemblages. The SES can be used for performing meta-analysis when multiple datasets are analyzed in a unified standard. Since this study only involves one dataset, we used pseudo *P-*value to test the significance as introduced below.

A pseudo *P*-value can be computed for A1 or A2 algorithms (Ma et al., 2019). Suppose *D*, the times when the number of *shared species* from 1,000 times of random re-sampling, does not exceed the number of *shared species observed*, then:


(2) $$\begin{array}{l} P=D/1000 \end{array}$$

**Figure 2:**
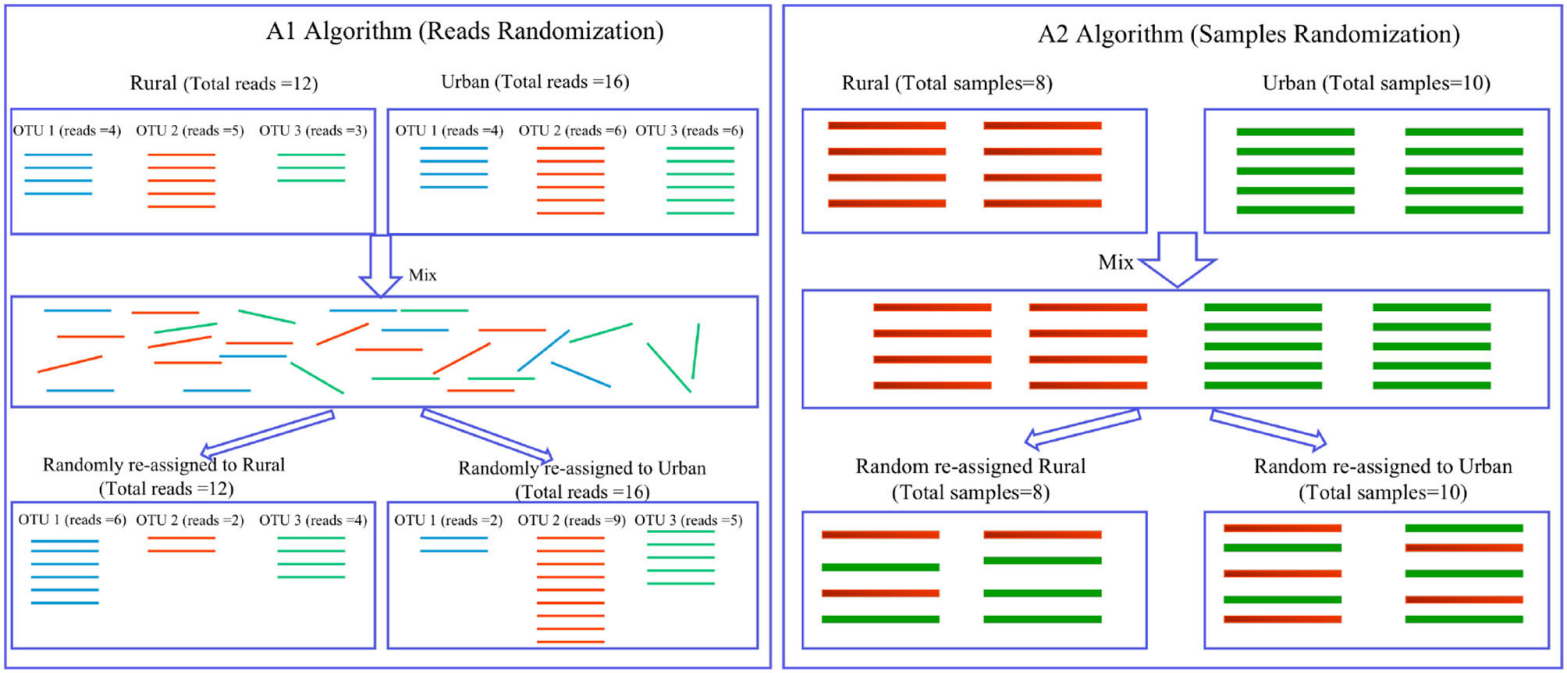
Diagrams illustrating the shared species analysis (SSA) with A1 and A2 algorithms (Ma et al., 2019): (*i*) A1 (the left) uses reads randomization, in which 16S-rRNA reads (*i.e.*, bacterial individuals) are represented with lines of various colors (different colors for different kinds of species or OTUs); the intermediate box contains mixed reads, without orders, but each read keeping track of its identity (OTU number or species name); 3 species or OTUs were assumed, and 12 and 16 reads were assumed in the rural and urban treatments, respectively. (*ii*) A2 (the right) uses samples randomization, in which the top left and right boxes contain 6 rural and 4 urban microbiome samples, respectively. The middle box contains mixed samples of 10 samples, pooled from the rural (6 samples) and urban (4 samples) groups. In the bottom, the pooled samples were reassigned randomly to the rural and urban groups again, but the samples were randomly mixed here. Rural *vs*. urban lifestyles were used as examples of treatments, and the algorithms are applicable to any pairs of cohorts.

If pseudo *P* < 0.05, then one can conclude that the difference in shared species cannot be attributed to random effects alone, and lifestyle is very likely to exert a significant effect on the number of shared species. An illustrative diagram is presented in Figure 2 to explain A1 and A2 algorithms, and detailed description of them is referred to Ma et al. (2019).

In addition, we computed the percentage of reduction in shared OTUs as follows:


(3) $$\begin{array}{l} R\left(\%\right)=\left[SOTU_{obs}-mean\left(SOTU_{sim}\right)\right]/\left(SOTU_{obs}\right), \end{array}$$

which measures the change (almost always reduction) of shared OTUs between two treatments.

In addition, from shared species (OTU) analysis, one can obtain two lists of unique species in either of the two treatments in a pair-wise comparison, as well as the list of shared species.

### Diversity Analysis in Hill Numbers

Alpha diversity refers to the diversity within a community, specified region, or ecosystem and is usually measured with some kind of entropy function of species-relative abundance and evenness. Scientists have proposed many diversity metrics (indexes), and diversity measures may have different ways of expression and reflect different perceptions of true diversity. For example, species richness, Shannon entropy (Shannon, 1948), and Simpson index (Simpson, 1949) are among the most commonly used and are based on abundance information and weighted for rare, moderately abundant and highly abundant species, respectively (Koh, 2018). Nevertheless, most of the diversity metrics, including these three, do not have a commonly comparable “unit.” In other words, they are hardly comparable. There is an exception – the Hill numbers, which were introduced by Hill (1973) as biodiversity metrics based on Renyi's general entropy and rediscovered by Chao et al. (2012, 2014) in consideration of the advantages of Hill numbers and insufficient early attention to Hill numbers. The Hill number is considered the most appropriate metric currently available for measuring alpha diversity (Ellison, 2010; Chao et al., 2012, 2014; Ma and Li, 2018), which is defined as:


(4) $${}^{q}H={\left(\sum_{i=1}^{S}p_{i}^{q}\right)}^{1/\left(1-q\right)\;}$$

where *S* is the number of species, *p*_*i*_ is the relative abundance of species *i*, and *q* is the order number of diversity. Hill number with *q* = 0 corresponds to species richness, in which species abundances do not weigh in. Hill numbers at *q* = 1 and *q* = 2 correspond, respectively to algebraic transformations of Shannon entropy and Simpson's index. In general, ^*q*^*H*, diversity of order *q*, represents for the diversity of a community with *x* = ^*q*^*H* equivalent species, which is characterized by the weighing scheme specified by *q*. With the increase of *q*, the Hill number index is increasingly weighted by the relative abundances of more abundant species and less weighted by rare species (Chao et al., 2014). When *q* = 0, rare species (lower abundances), common species, and even dominant species (higher abundances) weigh the Hill number equally. When *q* = 1, the Hill number is weighted most by common species and is a function of Shannon entropy. When *q* = 2, the Hill number is weighted most by dominant species, and it is a function of Simpson index. When *q* = 3, the Hill number is weighted more by even more dominant species. In all diversity orders (*q*), Hill numbers are the number of equivalent species characterized by a *q*-specific weighing scheme. Therefore, although Hill numbers at *q* = 0, 1, and 2 are special functions of species richness, Shannon entropy and Simpson index, respectively, the functional relationships transform as the number of species equivalents, and, therefore, the Hill number at each diversity order (*q*) has a *q*-specific unit. This species equivalence makes the comparisons of Hill numbers with rank-sum-based Wilcoxon test, particularly suitable for comparing two paired treatments. In the present study, we used Wilcoxon test to perform pair-wise comparisons of the microbiome samples collected from Chinese individuals of different ethnic identities and lifestyles (rural *vs*. urban).

### The Stochastic Neutrality Analysis Framework

Ning et al. (2019) mathematical framework for stochasticity analysis is based on the notion that deterministic processes should drive ecological communities more similar or dissimilar than null expectation. They established sophisticated computational procedures to implement a null model for quantifying stochasticity. A key metric in their framework is the application of RuŽička (1958) similarity metrics, which is a generalization of Jaccard binary similarity coefficient.

Let *C*_*ij*_ be the observed similarity between a pair of communities (*i* and *j*):


(5) $$C_{ij}=\frac{\sum_{S}\min(p_{k}^{i},p_{k}^{j})}{\sum_{S}\max(p_{k}^{i},p_{k}^{j})}$$

where *S* is the number of species and $p_{k}^{i}$ and $p_{k}^{j}$ are the relative abundance of *k-*th species in the *i-*th and *j-*th community.

Further assume that there exist *m* local communities in a metacommunity, and let *C*_*ij*_ be the observed similarity between the *i-*th local community and the *j-*th local community in the metacommunity. Let *E*_*ij*_ be the null-expected similarity between the *i-*th community and the *j-*th community in one simulated metacommunity, and let $\bar{E_{ij}}$be the average of the null-expected similarity between the *i-*th and the *j-*th communities in 1,000 simulated metacommunities. In the above-described setting, there exist two possibilities when assessing the community stochasticity. One possibility (type A) is that deterministic processes can drive communities more similar, in which *C*_*ij*_ > $\bar{E_{ij}}$, and the stochasticity ratio (*SR*, for Type A) is


(6) $$\begin{array}{l} SR_{ij}^{A}=\frac{\bar{E_{ij}}}{C_{ij}}. \end{array}$$

Another possibility (Type B) is that deterministic processes drive communities more dissimilar, in which *C*_*ij*_ < $\bar{E_{ij}}$, and the stochasticity ratio (*SR*, for Type B) is


(7) $$\begin{array}{l} SR_{ij}^{B}=\frac{1-\bar{E_{ij}}}{1-C_{ij}}. \end{array}$$

The stochasticity ratio in the whole metacommunity is weighted average in the form of:


(8) $$\begin{array}{l} SR=\frac{\sum_{ij}^{n^{A}}SR_{ij}^{A}+\sum_{ij}^{n^{B}}SR_{ij}^{B}}{n^{A}+n^{B}}, \end{array}$$

where *n*^*A*^ and *n*^*B*^ are the numbers of the pair-wise similarities of type A and type B, respectively, *SR* is the *strength of stochasticity* in the community assembly, and ranges from 0 to 1 (100%). If the community assembly is totally deterministic without any stochasticity, then *SR* should be 0%; otherwise, *SR* should be 100% without any determinism. There is a technical issue with the above quantification of SR: When expected stochasticity is very low, *SR* could overestimate stochasticity. To overcome this issue, Ning et al. (2019) developed so-termed normalized stochasticity ratio (*NSR*), which enjoys higher precision than the *SR*. The *NSR* has the same principle as *SR*, but of more sophisticated computational formula that is omitted here.

In the present article, we used the *NSR* to determine the stochasticity or non-stochasticity [1–*NSR*] level within pairs of communities. Specifically, we computed an NSR value for each pair of communities that constitute a metacommunity based on the comparison of the metacommunity with 1,000 simulated neutral metacommunities as explained previously. For each treatment (*e.g*., *N* microbiome samples from *N* individual subjects of same ethnicity, one sample from each individual subject), there are *Combination* (*N*, 2) pairs of community samples, and one NSR value can be computed for each of the pairs. We then computed the arithmetic mean or average of these NSR-values to represent the stochasticity level of the treatment under analysis, or, simply, the NSR of the treatment. But we reiterate that the NSR is computed for one metacommunity or a pair of two local communities, and that the NSR for a treatment (= cohort or population) is the mean NSR of all possible pair-wised metacommunities within the treatment.

Since the population (= treatment or cohort) level NSR is averaged from *Combination* (*N*, 2) pairs (metacommunities) of the local communities within population, one can compare two populations (*e.g*., urban *vs*. rural populations) with non-parametric Wilcoxon test, which should reveal the effects of lifestyle on the stochasticity. The *complement* of stochasticity or *non-stochasticity* = (1–*NSR*) can be used to gauge the deterministic niche selection level within a metacommunity.

Ultimately, the purpose for estimating the stochasticity (non-stochasticity) in this study is to shed light on the *mechanism* of diversity or composition changes, that is, whether or not their changes are driven by stochastic neutral drifts or deterministic niche differentiations (such as the selection effects of ethnicity and/or lifestyles). In addition, we also evaluated the selection (stochasticity) level at different taxon levels from phylum to species of the gut microbiome.

## Results and Discussion

### Detecting the Effects of *Lifestyles* on the Gut Microbiomes With SSA and Diversity Analysis

We investigated the effects of lifestyles (rural vs. urban) on community structures of the Chinese gut microbiomes by applying the SSA and diversity analysis under the design Scheme 1A,B as illustrated in Figure 1. Tables 1, 2 display the tests results from schemes 1A,B, respectively. The left side of Tables 1, 2 exhibited the *P*-values from SSA, and the right side exhibited the *P*-values from diversity analysis (Hill numbers at *q* = 0–3). To save page space, Tables 1, 2 here contain brief and necessary information only, and more detailed results from schemes 1A,B are provided in the online Supplementary Tables (Supplementary Tables 1–3). Besides *P*-value, reduction of shared species (Equation 3) between treatments is also computed and displayed in the online Supplementary Tables. Supplementary Table 9 exhibits one example of the lists of shared and unique OTUs at the phylum level.

**Table 1:** The results(*P*-values) of the *shared species analyses* (SSA) and Wilcoxon tests for the differences in Chinese gut microbiome *diversity* (Hill numbers) and the means of the *Non-Stochasticity* with Scheme-1A (*i.e*., comparing rural *vs*. urban lifestyles under same ethnicity).

**Taxon**	**Ethnicity**	***P*** **-value of shared species analyses (SSA)**		***P*** **-value from diversity analysis**				**Non-Stochasticity** **=(** ***1–NSR*** **)**	
		**Reads randomization**	**Samples randomization**	***q* = 0**	***q* = 1**	***q* = 2**	***q* = 3**	**Rural**	**Urban**
Phylum	Bai	0.057	0.409	0.360	0.465	0.530	0.564	0.378	0.416
	Han	0.283	0.567	0.094	0.201	0.172	0.172	0.430	0.409
	Kazakh	0.180	0.698	0.394	0.110	0.110	0.110	0.384	0.657
	Mongol	0.016	0.289	0.000	0.001	0.007	0.010	0.332	0.522
	Tibetan	0.000	0.088	0.826	0.112	0.090	0.118	0.615	0.659
	Uyghur	0.203	0.614	0.430	0.219	0.219	0.193	0.656	0.424
	Zhuang	0.165	0.698	0.312	0.270	0.151	0.133	0.497	0.464
	(%) With significant difference | or mean (Std Error)	28.57% (2/7)	0.0%	14.29%	14.29%	14.29%	14.29%	0.470 (0.047)	0.507 (0.042)
Family	Bai	0.000	0.066	0.756	0.990	1.000	0.929	0.628	0.607
	Han	0.003	0.923	0.429	0.378	0.352	0.378	0.680	0.681
	Kazakh	0.000	0.591	0.867	0.393	0.262	0.235	0.669	0.821
	Mongol	0.000	0.000	0.000	0.000	0.001	0.005	0.499	0.737
	Tibetan	0.000	0.826	0.164	0.472	0.384	0.294	0.785	0.788
	Uyghur	0.000	0.097	0.088	0.862	0.808	0.702	0.757	0.643
	Zhuang	0.000	0.339	0.190	0.520	0.534	0.593	0.704	0.739
	(%) With significant difference | or mean (Std Error)	100% (7/7)	14.29% (1/7)	14.29%	14.29%	14.29%	14.29%	0.675 (0.035)	0.717 (0.029)
Genus	Bai	0.000	0.046	0.517	0.599	0.673	0.691	0.688	0.659
	Han	0.000	0.499	0.700	0.719	0.946	0.761	0.736	0.733
	Kazakh	0.000	0.326	0.973	0.556	0.471	0.431	0.719	0.847
	Mongol	0.000	0.000	0.000	0.000	0.001	0.001	0.545	0.773
	Tibetan	0.000	0.859	0.273	0.271	0.201	0.167	0.808	0.795
	Uyghur	0.000	0.094	0.118	0.862	0.917	0.972	0.781	0.685
	Zhuang	0.000	0.140	0.296	0.719	0.974	0.870	0.753	0.773
	(%) With significant difference | or mean (Std Error)	100% (7/7)	28.57% (2/7)	14.29%	14.29%	14.29%	14.29%	0.719 (0.033)	0.752 (0.025)
Species	Bai	0.000	0.054	0.844	0.885	0.519	0.703	0.705	0.714
	Han	0.000	0.564	0.896	0.655	0.984	0.803	0.785	0.768
	Kazakh	0.000	0.334	0.845	1.000	1.000	0.845	0.757	0.869
	Mongol	0.000	0.000	0.000	0.000	0.000	0.000	0.632	0.805
	Tibetan	0.000	0.203	0.005	0.005	0.006	0.005	0.849	0.832
	Uyghur	0.000	0.055	0.058	0.602	0.508	0.508	0.825	0.74
	Zhuang	0.000	0.034	0.113	0.520	0.102	0.060	0.798	0.807
	(%) With significant difference | or mean (Std Error)	100% (7/7)	28.57% (2/7)	28.57%	28.57%	28.57%	28.57%	0.764 (0.028)	0.791 (0.020)

**Table 2:** The results (*P*-values) of shared species analyses (SSA) and diversity analysis and the means of *non-stochasticity* of the Chinese gut microbiomeunder design Scheme-1B (*i.e*., comparing rural *vs*. urban lifestyles, with all 7 ethnic groups combined for each lifestyle).

**Taxon**	***P*** **-value of shared species analyses**		***P*** **-value from diversity analysis**				**#Non-stochasticity** **= (1–NSR)**	
	**A1 (reads randomization)**	**A2 (samples randomization)**	***q* = 0**	***q* = 1**	***q* = 2**	**Rural**	**Rural**	**Urban**
Phylum	0.879	1.000	0.014	0.025	0.017	0.018	0.492	0.494
Family	0.000	0.007	0.010	0.175	0.480	0.725	0.701	0.724
Genus	0.000	0.002	0.012	0.258	0.470	0.625	0.746	0.765
Species	0.000	0.003	0.030	0.979	0.515	0.414	0.796	0.802
Mean (Std. Error)							0.684 (0.067)	0.696 (0.069)

*#Refer to Supplementary Table 10 for the detailed results of NSR (the normalized stochasticity ratio). Color values are significant differences for the comparisons*.

From Table 1, for the test results of Scheme 1A, we summarized the following findings:

(*i*) The SSA is far more sensitive than diversity analysis in detecting the effects of lifestyles across all ethnic groups and/or taxon levels. Particularly, the A1 algorithm (with reads randomization) detected 100% differences between the rural and urban lifestyles across all 7 ethnic groups and 4 taxon levels, except for the phylum level at which ~28% of differences were detected. The SSA indicates that Chinese people living rural and urban lifestyles experience significant differences in their gut community species compositions, as shown by the lower-than-expected numbers of shared OTUs between both the lifestyles.

(*ii*) The diversity analysis based on Wilcoxon tests of the Hill numbers indicates that the differences between urban and rural lifestyles were only significant across 7 ethnic groups in only ~14–28% of pair-wise ethnic comparisons. This level of difference is far less significant than the previously summarized finding from SSA that is 100% (except for the phylum level of 28%) as in (*i*).

(*iii*) At the phylum taxon level, the structural differences between rural and urban lifestyles, in terms of either SSA (28%) or diversity analysis (14%), are less significant than at other taxon levels. At the phylum level, only ethnic groups of Tibetans (*P* < 0.001) and Mongolians (*P* = 0.016) showed significant differences between rural and urban lifestyles. The Bai ethnic group with *P* = 0.057 is on the boundary of significance and needs further investigation regarding the effects of lifestyles.

In summary, in terms of SSA, lifestyles significantly affect the *community compositions* of Chinese gut microbiomes across species, genus, and family levels in all 7 ethnic groups (100%), except for the phylum level where the lifestyle effects are limited or partially significant (28%) in Tibetan and Mongolians but not significant in other 4 ethnic groups, and possibly significant in the Bai ethnic group. Nevertheless, in terms of diversity analysis, lifestyles have limited or partial effects on the gut microbiome diversity, only significant in ~14–28% of lifestyle comparisons.

From Table 2, for the test results of Scheme 1B, we summarized the following findings:

(*i*) With Scheme-1B, the samples from all 7 ethnic groups under the same lifestyle type were pooled together, and the total samples were only distinguished as two “big” samples of rural and urban lifestyles. Here, except for the phylum level (*P* = 0.879), the differences from the SSA between rural and urban lifestyles are significant at species, genus, and family levels (*P* < 0.001). The exhibited effects of lifestyles may include the confounding effects of ethnicity or they are the mixed effects of both lifestyles and ethnicities. Therefore, the finding here only confirms the previous finding from SSA based on Scheme 1A.
(*ii*) With Scheme-1B, the diversity analysis reveals interesting findings. At diversity *q* = 0, the differences between both lifestyles are significant across all four taxon levels (*P* = 0.010~0.030). This finding suggests that both species composition (indicated by SSA) and numbers (indicated by *species richness* or Hill number at *q* = 0) are significantly influenced by lifestyles.
(*iii*) The results from SSA and diversity analysis at the phylum taxon level appear puzzling. The SSA did not reveal significant difference (*P* = 0.879) between both the lifestyles at the phylum level under Scheme 1B; however, the diversity analysis did reveal significant differences (*P* = 0.010~0.030). In this particular case of the phylum level, the diversity analysis seems more sensitive than the SSA in detecting the structural changes of the Chinese gut microbiomes, which is contrary to the previous patterns. How should we explain this abnormality? One explanation can be that, at the phylum level, while the community composition of phyla is relatively constant, the abundances of different phyla are actually more deeply influenced, and, therefore, the diversity metrics are actually altered.

**Figure 3:**
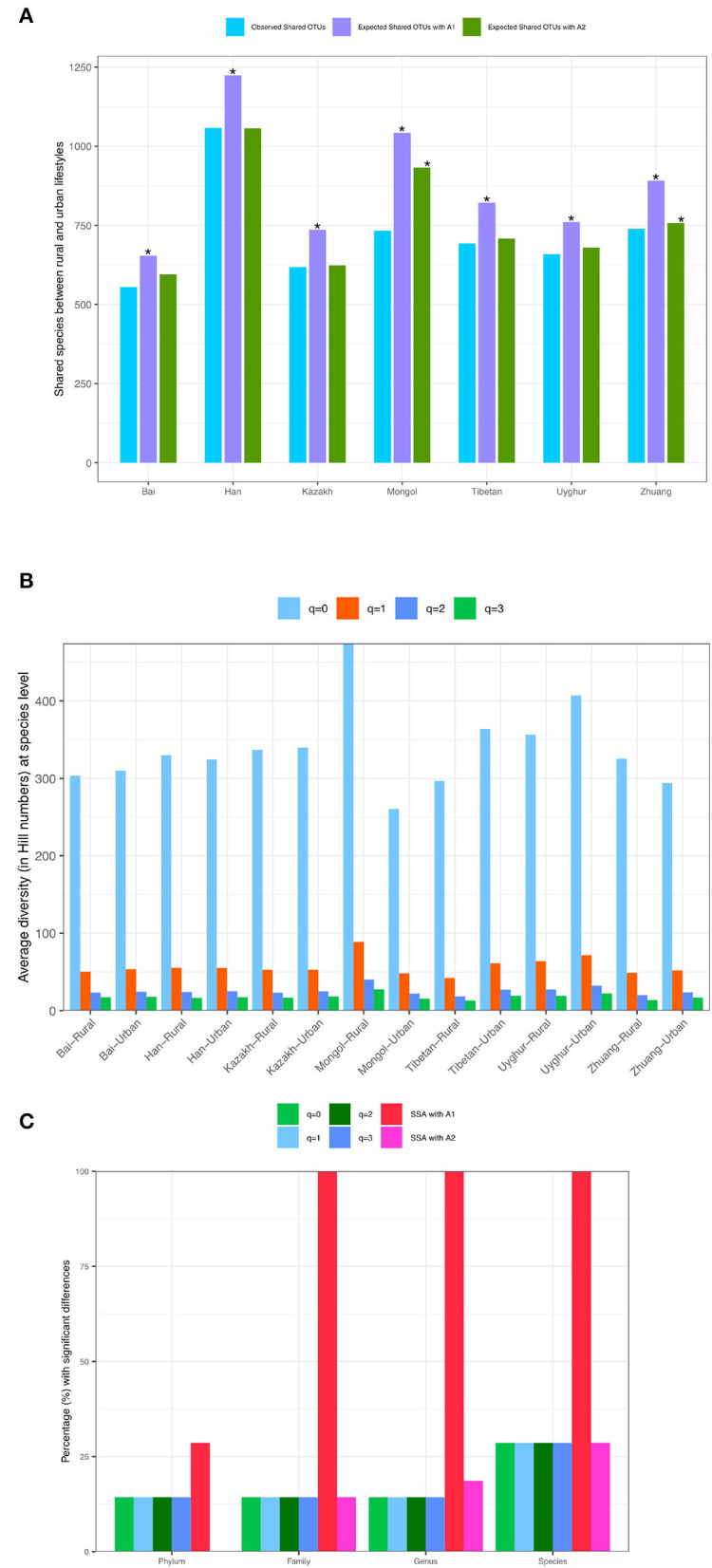
**(A)** The *observed* and *expected* numbers of *shared* species between *rural* and *urban* lifestyles for each of the seven major Chinese ethnic groups at the species taxonlevel, respectively (Scheme-1A). **(B)**. The *average* gut microbiome (the species level) diversity (in Hill numbers) for each of the 14 ethnicity-lifestyle combinations (Scheme-1A). **(C)** The percentage (%) with significant differences between the rural and urban lifestyles across 7 major ethnic groups based on SSA (shared species analysis, A1 and A2 algorithms) and diversity analysis (for *q* = 0–3) for the four taxon levels (phylum, family, genus, and species) (Scheme-1A). The “*” means there are significant differences for the comparison.

While Tables 1, 2 show the statistical test results (*P*-values) from Schemes 1A,B, Figure 3 illustrates the quantities of shared OTUs and diversity metrics (in Hill numbers). Figure 3A illustrates the *observed* and *expected* numbers of *shared* species between *rural* and *urban* lifestyles for each of the seven major Chinese ethnic groups, respectively, at the species taxon level based on the SSA with Scheme 1A. Figure 3B illustrates the average gut microbiome (the species level) diversity (in Hill numbers at *q* = 0–3) for each of the 14 ethnicity-lifestyle combinations (7 ethnic groups X 2 lifestyles). Figure 3C simply illustrates the test results presented in Table 1 in Scheme 1A, *i.e.*, the percentage (%) with significant differences between the rural and urban lifestyles across 7 major ethnic groups based on SSA (A1 and A2 algorithms) and diversity analysis (for *q* = 0 to 3) for the four taxon levels (phylum, family, genus, and species).

In summary, these findings confirmed significant effects of lifestyles on the community structures. In particular, the effects are 100% in terms of community compositions (SSA) across all but the phylum (the phylum level = 29%) levels, and 14–29% in terms of community diversity (Hill numbers) across all four taxon levels.

### Detecting the Effects of *Ethnicities* on the Gut Microbiomes With SSA and Diversity Analysis

We investigated the effects of ethnicities on community structures of the Chinese gut microbiomes by applying the SSA and diversity analysis under the design schemes 2A,B as illustrated in Figure 1. Tables 3, 4 display the test results from schemes 2A,B, respectively. The left side of Tables 3, 4 exhibited the *P*-values from SSA, and the right side exhibited the *P*-values from diversity analysis (Hill numbers at *q* = 0–3). To save page space, Tables 3, 4 here contain brief and necessary information only, and more detailed results from schemes 1A,B are provided in the online Supplementary Tables (Supplementary Tables 4–6).

**Table 3:** The results (percentages % with significant differences) of shared species analysis (SSA), diversity analysis, and *non-stochasticity* with design Scheme-2A (The pair-wise comparison of ethnic groups for rural and urban life styles, respectively, at four taxon levels).

**Taxon**	**Lifestyle**	**% With significant differences from shared species analysis**		**% With significant differences from diversity analysis**				**#The mean (std error) of non-stochasticity**	
		**A1: reads randomization**	**A2: samples randomization**	***q* = 0**	***q* = 1**	***q* = 2**	***q* = 3**	**Ethnicity-1**	**Ethnicity-2**
Phylum	Rural	47.62% (10/21)	4.76% (1/21)	42.86%	33.34%	38.10%	47.63%	0.421 (0.020)	0.520 (0.026)
	Urban	61.9% (13/21)	0.00%	33.34%	42.87%	33.34%	38.10%	0.499 (0.024)	0.516 (0.021)
Family	Rural	100% (21/21)	28.57% (6/21)	33.34%	47.62%	42.86%	42.86%	0.651 (0.017)	0.698 (0.020)
	Urban	100% (21/21)	14.29% (3/21)	38.10%	38.10%	38.10%	33.33%	0.703 (0.018)	0.730 (0.013)
Genus	Rural	100% (21/21)	42.86% (9/21)	28.57%	52.39%	33.34%	38.10%	0.301 (0.016)	0.737 (0.018)
	Urban	100% (21/21)	23.81% (5/21)	47.62%	23.81%	23.81%	14.29%	0.755 (0.015)	0.756 (0.010)
Species	Rural	100% (21/21)	61.9% (13/21)	42.86%	23.81%	14.29%	4.76%	0.743 (0.014)	0.786 (0.015)
	Urban	100% (21/21)	61.9% (13/21)	57.14%	28.57%	9.52%	4.76%	0.782 (0.013)	0.800 (0.009)

*#Refer to Supplementary Table 11 for the detailed results of NSR (the normalized stochasticity ratio)*.

**Table 4:** The results (percentages % with significant differences) of shared species analyses (SSA), diversity analysis, and *non-stochasticity* of the Chinese gut microbiomes under design Scheme-2B (*i.e*., the pair-wise ethnicity comparisons with urban and rural lifestyles combined for each comparison).

**Taxon**	**% With significant differences from shared species analysis**		**% With significant differences from diversity analysis**				**#The mean (std error) of non-stochasticity**	
	**A1: reads randomization**	**A2: samples randomization**	***q* = 0**	***q* = 1**	***q* = 2**	***q* = 3**	**Ethnicity-1**	**Ethnicity-2**
Phylum	76.19% (16/21)	14.29% (3/21)	47.62%	38.1%	38.1%	38.1%	0.460 (0.019)	0.514 (0.015)
Family	100% (21/21)	28.57% (6/21)	33.33%	47.62%	38.1%	42.86%	0.681 (0.014)	0.712 (0.011)
Genus	100% (21/21)	23.81% (5/21)	33.33%	57.14%	47.62%	47.62%	0.727 (0.011)	0.748 (0.010)
Species	100% (21/21)	52.38% (11/21)	23.81%	42.86%	28.57%	19.05%	0.763 (0.011)	0.790 (0.009)

*#Refer to Supplementary Table 12 for the detailed results of NSR (the normalized stochasticity ratio)*.

From Table 3, for the test results of Scheme 2A, we summarized the following findings:

(*i*) The SSA is far more sensitive than diversity analysis in detecting the effects of ethnicities on both rural and urban lifestyles, respectively. Particularly, the A1 algorithm (with reads randomization) detected 100% differences between pair-wise ethnic groups across both lifestyles and four taxon levels, except for the phylum level at which significant differences were detected in 48–62% of the pair-wise ethnic comparisons.
(*ii*) The diversity analysis based on Wilcoxon tests of the Hill numbers suggests that the differences between pair-wise ethnic groups were detected in 5–57% of the pair-wise ethnic comparisons. The patterns of ethnicity effects are similar in both rural and urban lifestyles.

In summary, the SSA is more powerful than the diversity analysis in detecting the effects of ethnicities on the community structures of Chinese gut microbiomes, given that SSA reveals community compositional changes and diversity reveals entropy changes of diversity.

From Table 4, for the test results of Scheme 2B, we summarized the following findings:

(*i*) When the lifestyles are ignored by pooling together the samples from both the rural and urban lifestyles for each of the 7 ethnic groups, the SSA still reveals 100% significant differences across all 4 taxon levels, except for the phylum level, at which the effects are partial (76%).
(*ii*) When the lifestyles are ignored, diversity analysis reveals partially significant difference across four taxon levels, and the percentages with significant differences are between 19% and 57%.

In summary, these findings confirmed significant effects of ethnicities on the community structures. In particular, the effects are 100% in terms of community compositions (SSA) across all but the phylum (the phylum level = 48–62%) levels, and 5–57% in terms of community diversity (Hill numbers) across all four taxon levels.

**Figure 4:**
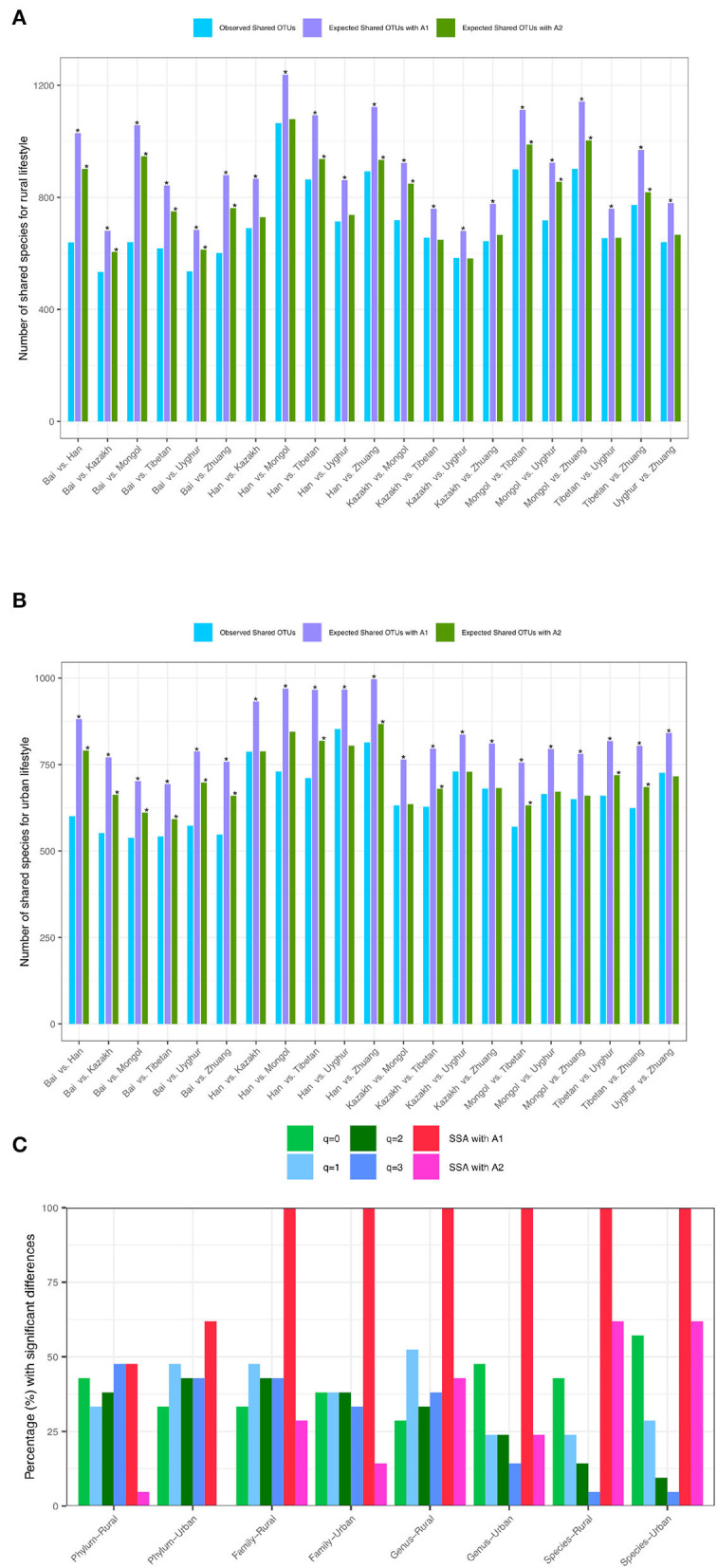
**(A)** The *observed* and*expected* numbers of shared species between pair-wise ethnic groups of the *rural* lifestyle at the species taxon level (with Scheme-2A). **(B)** The *observed* and *expected* numbers of shared species between pair-wise ethnic groups of the *urban* lifestyle at the species taxon level (with Scheme-2A). **(C)** The percentage (%) with significant differences between pair-wise ethnic groups based on SSA (shared species analysis for A1 and A2 algorithms) and diversity analysis (Hill numbers for *q* = 0 to 3) for each lifestyle at various taxon levels (phylum, family, genus, and species levels) (Scheme 2A). The “*” means there are significant differences for the comparison.

While Tables 3, 4 show the statistical test results (*P*-values) from Schemes 2A,B, Figure 4 illustrates the quantities of shared OTUs and diversity metrics (in Hill numbers). Figures 4A,B illustrate the *observed* and *expected* numbers of *shared* species between pair-wise ethnic groups for the rural and urban lifestyles, respectively, at the species-taxon level based on the SSA with Scheme 2A. Figure 4C simply illustrates the test results presented in Table 3 in Scheme 2A, *i.e.*, the percentage (%) with significant differences between pair-wise ethnic groups for each of the two lifestyles based on SSA (A1 and A2 algorithms) and diversity analysis (for *q* = 0–3) for the four taxon levels (phylum, family, genus, and species).

### Stochasticity (Non-Stochasticity) Analysis: Insights Into Selection Effects of Ethnicity (Lifestyle)

**Figure 5:**
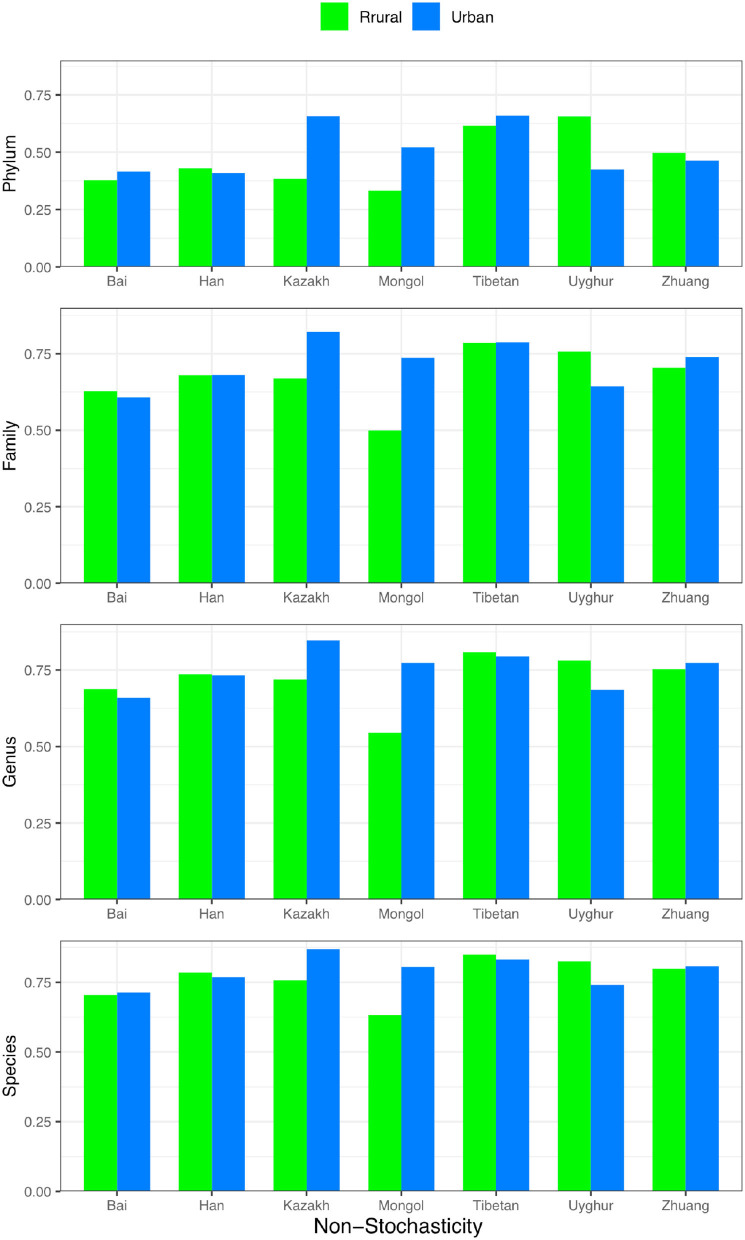
The non-stochasticity (1–*NSR*) (Y-axis) for each ethnic group (X-axis) with urban (blue) or rural (green) lifestyle, respectively; the non-stochasticity is displayed from species (bottom) to phylum (top).

For each of the four design schemes in Figure 1, we performed stochasticity analysis by applying Ning et al. (2019) and computed the NSR (the normalized stochasticity ratio) for each treatment (group) classified in Figure 1. In the last (rightmost) section of Tables 1–4, we tabulated the *non-stochasticity* (= 1–NSR) for each treatment (group) (also refer to Figure 5). In fact, what are listed in Tables 1–4 are the *averages* of the NSRs, as explained in the previous “material and methods” section. In the online Supplementary Tables (Supplementary Tables 9–12), more detailed results of the stochasticity analysis and *P*-values from Wilcoxon tests for testing the effects of ethnicity and/or lifestyle were displayed. Note that we reported *non-stochasticity* (= 1–NSR) in Tables 1–4 and Figure 5 in consideration that it directly indicates the level (strength) of deterministic selection forces, while NSR directly measures the level of stochasticity.

In Table 1 (Scheme 1A for comparing the rural *vs*. urban lifestyle under each of the 7 ethnic groups), the non-stochasticity increases from higher (phylum) to the lower taxonomic level (species) from ~0.5–0.8. This indicates that the non-stochasticity or selection forces are stronger at the lower taxon level, or the stochasticity is higher at the higher taxon level such as phylum. In summary, both deterministic selection forces (measured by non-stochasticity, such as ethnicities and lifestyles) and stochastic neutral drifts (measured by stochasticity) are in effects in all four taxon levels, but the selection becomes stronger and stochasticity becomes weaker from the phylum to the species level. Also, from the higher phylum to the lower species level, the balance between selection and stochasticity changes from nearly equal (~50 *vs*. 50%) to strongly imbalanced in favor of selection (~80 vs. 20%).

Supplementary Table 9 further lists more detailed NSR values, as well as the results from Wilcoxon tests. At the phylum level, ~40% of the pair-wise comparisons between rural and urban lifestyles across 7 ethnic groups showed significant differences (*P* < 0.05). The percentage declined to ~28% at other taxon levels (family, genus, and species). Furthermore, the stochasticity was usually significantly higher in rural lifestyle when the differences between urban and rural lifestyle are statistically significant. In other words, the selection was higher in urban lifestyle, which should have to do with the access to more industrialized food and, possibly, living environments. Therefore, from the lifestyle comparison based on the NSR, we may conclude that previously revealed differences between lifestyles by SSA and diversity analyses should, indeed, be attributed to stronger selection in urban lifestyle mechanistically.

It should be noted that, since each ethnic group may select group-specific taxa, similar to diversity analysis, there is no definite direction (increase or decrease) for the change caused by the selection effects of ethnicity. In other words, unlike lifestyle, in which urban lifestyle usually exerts stronger selection than rural lifestyle, the direction of selection in pair-wise comparison is uncertain, and the effects may cancel each other when cross-ethnicity comparisons are made. For this reason, the comparisons of NSR for the other three schemes are not informative, and we omitted their discussion here, although the results are provided in the online Supplementary Tables (Supplementary Tables 10–12). Overall, the results reported in Supplementary Tables 10–12 support the previous findings. That is, the selection force increases as the taxon level decreases from phylum to species, which simply means that species-level selection is more specifically targeted and is intuitively true in nature.

As to the difference between different treatments (urban vs. rural or pair-wise comparisons of ethnicities), Supplementary Tables 9–12 suggest that the differences range ~($\frac{1}{4}$–$\frac{3}{4}$). The low end ($\frac{1}{4}$) includes only one factor (lifestyle), and the high end ($\frac{3}{4}$) includes the effects of both ethnicity and lifestyle. Given that adding ethnicity (Supplementary Tables 10–12) increases the magnitude of difference approximately $\frac{1}{2}$, we postulate that the ethnicity effects appear stronger than lifestyle. This finding is also consistent with the differences previously revealed from SSA and diversity analysis.

## Conclusions and Discussion

From previous sections, we summarized the following findings: (*i*) Regarding the effects of lifestyles, SSA revealed 100% differences in terms of community compositions (SSA) across all but the phylum (the phylum level = 29%) levels, and 14–29% in terms of community diversity (Hill numbers) across all four taxon levels. (*ii*) Regarding the effects of ethnicities, SSA revealed 100% differences in terms of community compositions (SSA) across all but the phylum (the phylum level = 48–62%) levels, and 5–57% in terms of community diversity (Hill numbers) across all four taxon levels. (*iii*) The SSA can produce lists of unique and shared species (OTUs) as exemplified in Supplementary Table 8. (*iv*) The effects of ethnicity seem to be stronger than lifestyles in altering the community structures. (*v*) The stochasticity analysis reveals that the selection forces decline gradually from the phylum, the family, the genus through to the species level. At the phylum level, the balance between selection and stochasticity is almost balanced (~50 *vs*. 50%), and the balance is shifted to in favor of selection at the species level (<80 *vs*. 20%). Furthermore, the pair-wise difference between two treatments ranged from $\frac{1}{4}$ to $\frac{3}{4}$, approximately, with $\frac{1}{4}$ attributed to lifestyle and $\frac{1}{2}$ attributed to ethnicity. (*vi*) The community structures of the gut microbiomes are less variable (more stable) at the phylum level than at the other three levels of species, genus, and family. This is also supported by the stochasticity analysis, given that selection is weaker at the phylum level and, therefore, less variable.

The SSA is more powerful than standard diversity analysis in detecting the changes of community structures, given that the former considers the species identities and measures community compositions. The stochasticity suggests that the differences detected by SSA and diversity analysis can, indeed, be attributed to deterministic selection effects of ethnicities and/or lifestyles mechanistically.

In Zhang et al.'s (2015) original report (the dataset of which is reanalyzed in this study), theiranalysis based on canonical analysis of unweighted UniFrac principal coordinates clustered the subjects (microbiome samples) mainly by their ethnicities/geography and, less so, by lifestyles. They found that the structural differentiation of gut microbiota between the rural and urban cohorts varied greatly across geographical regions and ethnic groups. The Mongol ethnic group represented the most significant structural differentiation between the rural and urban cohorts in their study and also had the largest lifestyle-associated difference in Shannon diversity indices, as suggested by Student's *T-*test. The effects of lifestyle were most conspicuous within the Mongol and Zhuang ethnic groups, while this impact was marginally significant in the Tibetan cohorts, and the Uyghur and Kazakh showed the smallest lifestyle-associated distances (Zhang et al., 2015). Our study generally supports the original findings reported by Zhang et al. (2015); the contribution of our re-analysis lies in the demonstration of a systematic approach—shared species analysis (SSA)—that offers a more powerful approach to discerning the community structural (particularly community composition) changes beyond standard diversity analysis can deliver.

## Data Availability Statement

The original contributions presented in the study are included in the article/Supplementary Material, further inquiries can be directed to the corresponding author.

## Author Contributions

ZM designed the study, performed the study, and wrote the manuscript.

## Funding

This work was funded by National Natural Science Foundation of China (No. 31970116).

## Conflict of Interest

The author declares that the research was conducted in the absenceof any commercial or financial relationships that could be construed as a potential conflict of interest.

## Publisher's Note

All claims expressed in this article are solely those of the authors and do not necessarily represent those of their affiliated organizations, or those of the publisher, the editors and the reviewers. Any product that may be evaluated in this article, or claim that may be made by its manufacturer, is not guaranteed or endorsed by the publisher.
