# Supplementary material for: Shared Species Analysis, Augmented by Stochasticity Analysis, Is More Effective Than Diversity Analysis in Detecting Variations in the Gut Microbiomes
Source: Front Microbiol. 2022 Jul 19;13:914429. doi: 10.3389/fmicb.2022.914429 (PMC9343862; doi:10.3389/fmicb.2022.914429)
Supplement: Supplementary file 1 [file Data_Sheet_1.PDF]

Ma ZS (2022) Shared species analysis, augmented by stochasticity analysis, is more effective than diversity analysis in detecting variations in the gut microbiomes

List of Online Supplementary Tables S1-S12

Table S1. The results of shared species analyses (SSA) with design **Scheme 1A** (comparing the Rural *vs*. Urban lifestyles of the same ethnicity, *i.e*., ethnicity is fixed)

Table S2. The *mean* and *standard error* of the Hill numbers (diversity) for each of the 14 lifestyle-ethnicity combinatorial treatments

Table S3. The results of the shared species analyses (SSA) of Chinese gut microbiome with design **Scheme-1B** (Comparing the Rural *vs.* Urban lifestyles, with all 7 ethnic groups combined for each lifestyle)

Table S4. The *mean* and *standard error* of the Hill numbers (diversity) for each lifestyle with design **Scheme-1B** (Comparing the Rural *vs.* Urban lifestyles, with all 7 ethnic groups combined for each lifestyle)

Table S5. The results of shared species analyses (SSA) and Wilcoxon tests for the differences in diversity (Hill numbers) with design **Scheme-2A** (the pair-wise comparison among 7 ethnic groups, for rural and urban lifestyles, respectively)

Table S6. The results of shared species analyses (SSA) and Wilcoxon tests for the differences in diversity (Hill numbers) with design **Scheme-2B** (The pair-wise ethnicity comparisons among 7 ethnic groups, with urban & rural lifestyles pooled)

Table S7. The *mean* and *standard error* of the Hill numbers (diversity) for each ethnicity with both lifestyles pooled with design **Scheme-2B**.

Table S8. The unique phyla of and shared phyla between rural and urban lifestyle for each ethnic group, *i.e*., comparing the Rural *vs*. Urban lifestyles of same ethnic group

Table S9. The means of the *Normalized Stochastic Ratio* (*NSR*) of the Chinese gut microbiome with **Scheme-1A** (comparing the Rural *vs*. Urban lifestyles of same ethnicity), and the percentage with significant differences from Wilcoxon tests for the differences in the *NSR* between rural and urban groups (*P*-value=0.05)

Table S10. The means of the *Normalized Stochastic Ratio* (*NSR*) of the Chinese gut microbiome under design **Scheme-1B** (Comparing the Rural *vs.* Urban lifestyles, with all 7 ethnic groups combined for each lifestyle), and the percentage with significant difference from Wilcoxon test for the differences between rural and urban groups in *NSR* (*P*-value=0.05)

Table S11. The means of the *Normalized Stochastic Ratio* (*NSR*) of the Chinese gut microbiomes under design **Scheme-2A** (The pair-wise comparison of ethnic groups for rural and urban life styles, respectively, at four taxon

levels), and the percentage of ethnic pairs with significant differences in the NSR from the Wilcoxon (*P*-value=0.05)

Table S12. The means of the *Normalized Stochastic Ratio* (*NSR*) of the Chinese gut microbiomes under design **Scheme-2B** (The pair-wise ethnicity comparisons with urban & rural lifestyles combined for each comparison), and the percentage with significant difference from Wilcoxon test for the differences between different ethnicity groups in *NSR* (*P*-value=0.05)

Supplementary Tables (S1-12)

Table S1. The results of shared species analyses (SSA) with design **Scheme 1A** (comparing the Rural *vs*. Urban lifestyles of the same ethnicity, *i.e*., ethnicity is fixed)

Datasets	Rural vs. Urban	Observed Shared OTUs	Reads randomization			Samples randomization		
			Expected Shared OTUs	Reduction (%) of shared OTUs (eqn. 3)	*P*-value	Expected Shared OTUs	Reduction (%) of shared OTUs (eqn. 3)	*P*-value
Phylum	Bai	25	27.827	-11.308	0.057	25.719	-2.876	0.409
	Han	34	35.067	-3.138	0.283	34.274	-0.806	0.567
	Kazakh	25	26.691	-6.764	0.180	24.665	1.340	0.698
	Mongol	30	34.136	-13.787	0.016	31.266	-4.220	0.289
	Tibetan	22	28.233	-28.332	0.000	23.935	-8.795	0.088
	Uyghur	28	29.435	-5.125	0.203	28.072	-0.257	0.614
	Zhuang	29	30.617	-5.576	0.165	28.839	0.555	0.698
	Significant Difference (%)				28.6%(2/7)			0(0/7)
Family	Bai	134	160.609	-19.857	0.000	144.129	-7.559	0.066
	Han	203	213.382	-5.114	0.003	198.947	1.997	0.923
	Kazakh	130	142.537	-9.644	0.000	129.137	0.664	0.591
	Mongol	154	196.242	-27.430	0.000	180.806	-17.406	0.000
	Tibetan	146	163.343	-11.879	0.000	142.738	2.234	0.826
	Uyghur	131	144.691	-10.451	0.000	135.683	-3.575	0.097
	Zhuang	152	167.350	-10.099	0.000	153.701	-1.119	0.339
	Significant Difference (%)				100%(7/7)			14.3%(1/7)
Genus	Bai	266	326.506	-22.747	0.000	286.166	-7.581	0.046
	Han	403	444.823	-10.378	0.000	403.181	-0.045	0.499
	Kazakh	248	285.095	-14.958	0.000	250.993	-1.207	0.326
	Mongol	288	394.772	-37.074	0.000	354.430	-23.066	0.000
	Tibetan	295	338.598	-14.779	0.000	287.751	2.457	0.859
	Uyghur	262	290.848	-11.011	0.000	270.721	-3.329	0.094
	Zhuang	293	333.508	-13.825	0.000	298.843	-1.994	0.140
	Significant Difference (%)				100%(7/7)			28.6%(2/7)
Species	Bai	555	654.103	-17.856	0.000	595.425	-7.284	0.054
	Han	1058	1224.384	-15.726	0.000	1056.419	0.149	0.564
	Kazakh	618	735.762	-19.055	0.000	623.290	-0.856	0.334
	Mongol	733	1042.538	-42.229	0.000	932.553	-27.224	0.000
	Tibetan	693	821.681	-18.569	0.000	708.196	-2.193	0.203
	Uyghur	659	760.641	-15.424	0.000	679.356	-3.089	0.055
	Zhuang	739	890.633	-20.519	0.000	756.778	-2.406	0.034
	Significant Difference (%)				100%(7/7)			28.6%(2/7)

Table S2. The *mean* and *standard error* of the Hill numbers (diversity) for each of the 14 lifestyle-ethnicity combinatorial treatments

Datasets	Treatments	Statistics	*q*=0	*q*=1	*q*=2	*q*=3
Phylum	Bai-Rural	Mean	15.931	2.522	1.819	1.648
		Std. Err.	0.559	0.138	0.097	0.080
	Bai-Urban	Mean	16.571	2.581	1.874	1.718
		Std. Err.	0.709	0.132	0.122	0.116
	Han-Rural	Mean	16.636	2.506	1.787	1.622
		Std. Err.	0.428	0.100	0.079	0.070
	Han-Urban	Mean	15.787	2.321	1.624	1.475
		Std. Err.	0.419	0.079	0.052	0.042
	Kazakh-Rural	Mean	17.000	2.915	1.901	1.676
		Std. Err.	0.645	0.236	0.157	0.126
	Kazakh-Urban	Mean	16.231	3.410	2.366	2.073
		Std. Err.	0.752	0.245	0.181	0.149
	Mongol-Rural	Mean	19.500	3.314	2.183	1.912
		Std. Err.	0.465	0.105	0.064	0.055
	Mongol-Urban	Mean	14.955	2.722	1.931	1.727
		Std. Err.	0.381	0.132	0.093	0.076
	Tibetan-Rural	Mean	14.893	3.042	2.304	2.108
		Std. Err.	0.510	0.148	0.116	0.104
	Tibetan-Urban	Mean	15.133	2.702	2.022	1.854
		Std. Err.	0.593	0.200	0.148	0.126
	Uyghur-Rural	Mean	18.333	3.940	2.852	2.530
		Std. Err.	0.816	0.421	0.360	0.321
	Uyghur-Urban	Mean	19.417	3.172	2.099	1.840
		Std. Err.	0.763	0.148	0.113	0.098
	Zhuang-Rural	Mean	16.000	2.674	1.859	1.673
		Std. Err.	0.482	0.106	0.072	0.062
	Zhuang-Urban	Mean	15.364	2.574	1.808	1.633
		Std. Err.	0.533	0.191	0.159	0.140
	Total samples	Mean	16.554	2.885	2.030	1.821
		Std. Err.	0.575	0.170	0.130	0.112
Family	Bai-Rural	Mean	75.448	12.508	8.144	6.896
		Std. Err.	3.564	0.622	0.526	0.484
	Bai-Urban	Mean	71.071	12.551	8.116	6.805
		Std. Err.	2.198	0.564	0.593	0.591
	Han-Rural	Mean	77.273	12.513	8.174	6.940
		Std. Err.	2.464	0.552	0.418	0.365
	Han-Urban	Mean	74.787	12.255	7.866	6.653
		Std. Err.	2.169	0.457	0.376	0.348
	Kazakh-Rural	Mean	78.444	13.474	8.227	6.586
		Std. Err.	5.047	1.516	1.213	1.082
	Kazakh-Urban	Mean	78.462	13.860	9.234	7.904
		Std. Err.	4.364	1.199	1.078	0.983
	Mongol-Rural	Mean	100.192	17.955	11.534	9.564
		Std. Err.	3.370	0.925	0.787	0.726
	Mongol-Urban	Mean	64.136	12.415	8.280	6.998
		Std. Err.	1.550	0.622	0.482	0.451
	Tibetan-Rural	Mean	69.250	11.184	6.823	5.580
		Std. Err.	2.664	0.715	0.554	0.474
	Tibetan-Urban	Mean	76.600	12.015	7.731	6.508
		Std. Err.	3.112	0.988	0.843	0.766
	Uyghur-Rural	Mean	81.556	15.911	9.956	8.032
		Std. Err.	3.660	1.254	1.091	0.994
	Uyghur-Urban	Mean	91.000	15.855	10.282	8.648
		Std. Err.	3.572	0.770	0.727	0.713
	Zhuang-Rural	Mean	74.000	12.951	8.255	6.870
		Std. Err.	2.674	0.603	0.495	0.450
	Zhuang-Urban	Mean	69.500	12.287	7.761	6.441
		Std. Err.	2.324	0.620	0.499	0.448
	Total samples	Mean	77.266	13.409	8.599	7.173
		Std. Err.	3.052	0.815	0.692	0.634
Genus	Bai-Rural	Mean	139.138	21.583	12.418	9.903
		Std. Err.	6.785	1.137	0.734	0.613
	Bai-Urban	Mean	131.429	20.516	11.858	9.547
		Std. Err.	5.192	1.311	1.140	1.042
	Han-Rural	Mean	141.182	20.806	11.586	9.151

		Std. Err.	4.935	0.952	0.610	0.496
	Han-Urban	Mean	139.106	20.873	11.874	9.515
		Std. Err.	4.497	0.839	0.576	0.490
	Kazakh-Rural	Mean	147.556	23.260	14.151	11.658
		Std. Err.	11.286	2.266	1.637	1.425
	Kazakh-Urban	Mean	145.923	21.250	12.111	9.643
		Std. Err.	8.957	1.824	1.333	1.132
	Mongol-Rural	Mean	194.192	29.470	16.807	13.321
		Std. Err.	7.124	1.576	1.085	0.913
	Mongol-Urban	Mean	116.682	19.481	11.541	9.354
		Std. Err.	3.236	1.045	0.725	0.630
	Tibetan-Rural	Mean	124.964	16.356	8.664	6.784
		Std. Err.	5.636	1.288	0.838	0.672
	Tibetan-Urban	Mean	139.933	18.898	10.867	8.879
		Std. Err.	6.854	1.905	1.433	1.256
	Uyghur-Rural	Mean	155.556	25.620	14.345	11.191
		Std. Err.	5.718	2.334	1.761	1.506
	Uyghur-Urban	Mean	172.667	25.816	14.492	11.371
		Std. Err.	7.486	1.567	1.073	0.906
	Zhuang-Rural	Mean	137.750	20.374	11.772	9.473
		Std. Err.	5.777	1.210	0.860	0.740
	Zhuang-Urban	Mean	130.500	20.710	11.579	9.112
		Std. Err.	5.040	0.883	0.602	0.516
	Total samples	Mean	144.041	21.787	12.433	9.922
		Std. Err.	6.323	1.438	1.029	0.881
Species	Bai-Rural	Mean	303.517	50.242	23.424	17.434
		Std. Err.	11.507	3.051	1.651	1.191
	Bai-Urban	Mean	309.929	53.679	24.476	17.981
		Std. Err.	17.720	4.311	2.519	1.950
	Han-Rural	Mean	329.659	55.499	24.175	16.375
		Std. Err.	12.793	3.502	1.874	1.295
	Han-Urban	Mean	324.340	55.266	24.956	17.511
		Std. Err.	11.688	3.169	1.864	1.389
	Kazakh-Rural	Mean	336.778	52.926	23.168	16.568
		Std. Err.	28.835	7.253	3.864	2.791
	Kazakh-Urban	Mean	339.692	52.950	24.874	18.310
		Std. Err.	24.318	6.557	3.605	2.668
	Mongol-Rural	Mean	474.077	88.678	40.229	27.727
		Std. Err.	18.960	5.271	3.008	2.196
	Mongol-Urban	Mean	260.273	48.134	22.107	15.605
		Std. Err.	7.187	3.062	1.724	1.253
	Tibetan-Rural	Mean	296.571	42.108	18.381	13.114
		Std. Err.	14.097	4.186	2.298	1.646
	Tibetan-Urban	Mean	363.600	61.176	26.961	19.187
		Std. Err.	15.959	4.016	2.044	1.473
	Uyghur-Rural	Mean	356.444	64.070	27.432	19.107
		Std. Err.	13.404	7.147	4.751	3.546
	Uyghur-Urban	Mean	407.000	71.543	32.481	22.332
		Std. Err.	21.738	6.293	3.894	2.868
	Zhuang-Rural	Mean	325.250	49.022	20.101	13.713
		Std. Err.	15.289	3.412	1.671	1.138
	Zhuang-Urban	Mean	293.818	51.991	23.733	16.715
		Std. Err.	12.162	3.041	1.683	1.245
	Total samples	Mean	337.211	56.949	25.464	17.977
		Std. Err.	16.118	4.591	2.604	1.904

Table S3. The results of the shared species analyses (SSA) of Chinese gut microbiome with design **Scheme-1B** (Comparing the Rural *vs.* Urban lifestyles, with all 7 ethnic groups combined for each lifestyle)

Datasets	Observed Shared OTUs	Reads randomization			Samples randomization		
		Expected Shared OTUs	Reduction (%) of shared OTUs (eqn. 3)	*P*-value	Expected Shared OTUs	Reduction (%) of shared OTUs (eqn. 3)	*P*-value
Phylum	46	45.373	2.204	0.879	44.347	2.255	1.000
Family	270	297.604	0.336	0.000	281.347	0.355	0.007
Genus	576	638.753	0.157	0.000	595.518	0.168	0.002
Species	1525	1717.452	0.058	0.000	1557.802	0.064	0.003

Table S4. The *mean* and *standard error* of the Hill numbers (diversity) for each lifestyle with design **Scheme-1B** (Comparing the Rural *vs.* Urban lifestyles, with all 7 ethnic groups combined for each lifestyle)

Dataset	Treatments	The Hill numbers (diversity)				
		Statistics	*q*=0	*q*=1	*q*=2	*q*=3
Phylum	Rural	Mean	16.686	2.844	2.012	1.81
		Std. Err.	0.229	0.062	0.046	0.04
	Urban	Mean	15.945	2.653	1.869	1.684
		Std. Err.	0.229	0.061	0.045	0.038
Family	Rural	Mean	78.982	13.423	8.571	7.14
		Std. Err.	1.414	0.331	0.256	0.227
	Urban	Mean	73.869	12.73	8.246	6.95
		Std. Err.	1.156	0.271	0.226	0.209
Genus	Rural	Mean	146.917	21.861	12.358	9.817
		Std. Err.	2.955	0.588	0.387	0.32
	Urban	Mean	137.131	20.841	11.911	9.532
		Std. Err.	2.418	0.475	0.334	0.286
Species	Rural	Mean	343.089	56.882	25.097	17.541
		Std. Err.	7.442	1.984	1.045	0.736
	Urban	Mean	320.876	55.284	25.115	17.791
		Std. Err.	6.466	1.611	0.908	0.67

Table S5. The results of shared species analyses (SSA) and Wilcoxon tests for the differences in diversity (Hill numbers) with design **Scheme-2A** (the pair-wise comparison among 7 ethnic groups, for rural and urban lifestyles, respectively)

Taxon	Group	Treatment	Observed Shared OTUs	Reads randomization			Samples randomization			P-value of Wilcoxon test for the Hill numbers			
				Expected Shared OTUs	Reduction (%) of shared OTUs (eqn. 3)	*P*-value	Expected Shared OTUs	Reduction (%) of shared OTUs (eqn. 3)	*P*-value	*q=0*	*q=1*	*q=2*	*q=3*
Phylum	Rural	Bai *vs*. Han	31	33.249	3.008	0.061	31.944	3.130	0.334	0.142	0.969	0.750	0.650
		Bai *vs*. Kazakh	23	27.052	3.697	0.013	24.421	4.095	0.329	0.103	0.133	0.589	0.686
		Bai *vs*. Mongol	32	34.381	2.909	0.111	31.665	3.158	0.753	0.000	0.000	0.001	0.003
		Bai *vs*. Tibetan	26	29.362	3.406	0.008	27.469	3.640	0.158	0.252	0.005	0.001	0.001
		Bai *vs*. Uyghur	25	27.361	3.655	0.112	24.890	4.018	0.612	0.021	0.002	0.013	0.016
		Bai *vs*. Zhuang	29	30.371	3.293	0.271	28.279	3.536	0.836	0.595	0.185	0.333	0.409
		Han *vs*. Kazakh	25	29.552	3.384	0.011	27.567	3.628	0.127	0.784	0.116	0.331	0.368
		Han *vs*. Mongol	34	35.447	2.821	0.156	33.838	2.955	0.672	0.000	0.000	0.000	0.000
		Han *vs*. Tibetan	28	33.107	3.021	0.000	31.439	3.181	0.006	0.010	0.003	0.000	0.000
		Han *vs*. Uyghur	28	29.420	3.399	0.277	27.915	3.582	0.644	0.105	0.001	0.006	0.008
		Han *vs*. Zhuang	32	33.926	2.948	0.090	31.540	3.171	0.785	0.339	0.213	0.185	0.153
		Kazakh *vs*. Mongol	26	29.613	3.377	0.041	27.729	3.606	0.231	0.008	0.160	0.073	0.042
		Kazakh *vs*. Tibetan	22	26.373	3.792	0.002	23.646	4.229	0.215	0.022	0.821	0.080	0.030
		Kazakh *vs*. Uyghur	24	29.224	3.422	0.000	24.440	4.092	0.508	0.244	0.113	0.113	0.113
		Kazakh *vs*. Zhuang	25	26.741	3.740	0.209	24.791	4.034	0.678	0.357	0.462	0.984	0.921
		Mongol *vs*. Tibetan	29	32.656	3.062	0.003	31.140	3.211	0.099	0.000	0.053	0.870	0.199
		Mongol *vs*. Uyghur	27	30.740	3.253	0.042	28.339	3.529	0.314	0.235	0.160	0.184	0.210
		Mongol *vs*. Zhuang	32	34.469	2.901	0.113	31.579	3.167	0.724	0.000	0.000	0.001	0.010
		Tibetan *vs*. Uyghur	23	26.735	3.740	0.010	23.429	4.268	0.492	0.004	0.053	0.240	0.302
		Tibetan *vs*. Zhuang	27	28.615	3.495	0.102	25.902	3.861	1.000	0.129	0.117	0.006	0.002
		Uyghur *vs*. Zhuang	26	27.485	3.638	0.271	24.772	4.037	0.913	0.028	0.020	0.049	0.054
	Significant Difference (%)					47.6%(10/21)			4.8%(1/21)	42.86%	33.33%	38.10%	47.62%
	Urban	Bai *vs*. Han	27	29.364	3.406	0.123	27.971	3.575	0.383	0.373	0.076	0.055	0.045
		Bai *vs*. Kazakh	24	25.819	3.873	0.120	23.567	4.243	0.795	0.883	0.005	0.054	0.094
		Bai *vs*. Mongol	25	27.259	3.669	0.063	24.557	4.072	0.766	0.068	0.553	0.642	0.642
		Bai *vs*. Tibetan	24	26.080	3.834	0.118	23.363	4.280	0.828	0.140	0.983	0.477	0.533
		Bai *vs*. Uyghur	24	28.213	3.544	0.000	26.577	3.763	0.056	0.014	0.013	0.212	0.274
		Bai *vs*. Zhuang	26	27.701	3.610	0.106	26.307	3.801	0.550	0.218	0.553	0.281	0.227
		Han *vs*. Kazakh	26	29.512	3.388	0.045	27.480	3.639	0.287	0.574	0.000	0.000	0.000
		Han *vs*. Mongol	28	31.929	3.132	0.015	29.523	3.387	0.240	0.207	0.008	0.002	0.001
		Han *vs*. Tibetan	26	31.891	3.136	0.000	28.666	3.488	0.112	0.370	0.065	0.010	0.005
		Han *vs*. Uyghur	29	32.210	3.105	0.047	29.168	3.428	0.565	0.000	0.000	0.000	0.000
		Han *vs*. Zhuang	31	31.906	3.134	0.366	30.823	3.244	0.669	0.594	0.231	0.296	0.334
		Kazakh *vs*. Mongol	25	26.904	3.717	0.140	24.579	4.069	0.773	0.145	0.020	0.026	0.038
		Kazakh *vs*. Tibetan	24	26.722	3.742	0.053	24.327	4.111	0.545	0.217	0.029	0.108	0.254
		Kazakh *vs*. Uyghur	25	29.561	3.383	0.000	27.377	3.653	0.086	0.010	0.295	0.225	0.152
		Kazakh *vs*. Zhuang	26	29.403	3.401	0.031	27.386	3.652	0.291	0.333	0.003	0.004	0.005
		Mongol *vs*. Tibetan	24	28.501	3.509	0.000	24.454	4.089	0.509	0.900	0.680	0.614	0.435
		Mongol *vs*. Uyghur	24	29.814	3.354	0.000	26.152	3.824	0.116	0.000	0.013	0.127	0.327
		Mongol *vs*. Zhuang	26	30.086	3.324	0.000	27.222	3.673	0.282	0.439	0.268	0.085	0.055
		Tibetan *vs*. Uyghur	25	28.918	3.458	0.001	26.861	3.723	0.121	0.000	0.025	0.683	0.981

		Tibetan *vs.* Zhuang	25	28.466	3.513	0.005	26.592	3.761	0.202	0.605	0.572	0.080	0.056
		Uyghur *vs.* Zhuang	27	28.943	3.455	0.085	27.767	3.601	0.392	0.000	0.002	0.011	0.011
	Significant Difference (%)					61.9%(12/21)			0(0/21)	33.33%	42.865	33.33%	38.10%
Family	Rural	Bai *vs.* Han	185	202.200	0.495	0.000	184.967	0.541	0.554	0.404	0.775	0.889	0.933
		Bai *vs.* Kazakh	132	154.600	0.647	0.000	138.575	0.722	0.257	0.381	0.457	0.919	0.589
		Bai *vs.* Mongol	192	210.727	0.475	0.000	193.129	0.518	0.414	0.000	0.000	0.001	0.005
		Bai *vs.* Tibetan	165	183.689	0.544	0.000	166.056	0.602	0.417	0.350	0.141	0.070	0.045
		Bai *vs.* Uyghur	138	156.971	0.637	0.000	139.839	0.715	0.439	0.131	0.012	0.162	0.360
		Bai *vs.* Zhuang	165	182.890	0.547	0.000	166.217	0.602	0.401	0.872	0.541	0.922	0.965
		Han *vs.* Kazakh	133	159.163	0.628	0.000	148.297	0.674	0.028	0.758	0.381	0.701	0.297
		Han *vs.* Mongol	203	216.967	0.461	0.000	201.519	0.496	0.666	0.000	0.000	0.000	0.002
		Han *vs.* Tibetan	166	196.093	0.510	0.000	179.671	0.557	0.000	0.073	0.091	0.050	0.023
		Han *vs.* Uyghur	144	160.310	0.624	0.000	149.700	0.668	0.260	0.302	0.034	0.192	0.448
		Han *vs.* Zhuang	171	194.417	0.514	0.000	177.683	0.563	0.069	0.496	0.804	0.894	0.679
		Kazakh *vs.* Mongol	137	172.968	0.578	0.000	164.736	0.607	0.001	0.004	0.013	0.031	0.046
		Kazakh *vs.* Tibetan	125	140.827	0.710	0.000	127.430	0.785	0.339	0.215	0.116	0.286	0.373
		Kazakh *vs.* Uyghur	119	137.548	0.727	0.000	120.821	0.828	0.316	0.595	0.258	0.340	0.387
		Kazakh *vs.* Zhuang	128	145.855	0.686	0.000	135.596	0.737	0.105	0.418	0.512	0.796	0.592
		Mongol *vs.* Tibetan	174	205.678	0.486	0.000	190.857	0.524	0.000	0.000	0.000	0.000	0.000
		Mongol *vs.* Uyghur	142	176.261	0.567	0.000	166.498	0.601	0.001	0.005	0.128	0.210	0.210
		Mongol *vs.* Zhuang	170	203.217	0.492	0.000	186.629	0.536	0.001	0.000	0.000	0.002	0.005
		Tibetan *vs.* Uyghur	126	144.312	0.693	0.000	128.479	0.778	0.337	0.019	0.004	0.023	0.044
		Tibetan *vs.* Zhuang	151	171.752	0.582	0.000	153.511	0.651	0.239	0.326	0.055	0.068	0.062
		Uyghur *vs.* Zhuang	132	147.920	0.676	0.000	135.549	0.738	0.275	0.089	0.044	0.142	0.370
	Significant Difference (%)					100%(21/21)			28.6%(6/21)	33.33%	47.62%	42.86%	42.86%
	Urban	Bai *vs.* Han	141	170.375	0.587	0.000	156.623	0.638	0.005	0.548	0.859	0.793	0.926
		Bai *vs.* Kazakh	126	141.645	0.706	0.000	127.492	0.784	0.357	0.233	0.185	0.239	0.239
		Bai *vs.* Mongol	124	133.541	0.749	0.000	121.449	0.823	0.832	0.005	0.835	0.642	0.597
		Bai *vs.* Tibetan	128	145.183	0.689	0.000	129.419	0.773	0.363	0.205	0.621	0.621	0.683
		Bai *vs.* Uyghur	127	144.276	0.693	0.000	135.691	0.737	0.008	0.000	0.002	0.041	0.085
		Bai *vs.* Zhuang	134	145.870	0.686	0.000	132.836	0.753	0.650	0.454	0.911	0.987	0.835
		Han *vs.* Kazakh	165	174.860	0.572	0.008	160.436	0.623	0.736	0.518	0.059	0.079	0.099
		Han *vs.* Mongol	149	181.615	0.551	0.000	167.284	0.598	0.000	0.001	0.632	0.360	0.544
		Han *vs.* Tibetan	155	187.347	0.534	0.000	163.567	0.611	0.094	0.570	0.757	0.922	0.858
		Han *vs.* Uyghur	161	177.730	0.563	0.000	159.223	0.628	0.611	0.001	0.000	0.007	0.026
		Han *vs.* Zhuang	164	183.837	0.544	0.000	171.573	0.583	0.077	0.168	0.641	0.995	0.793
		Kazakh *vs.* Mongol	135	145.386	0.688	0.001	130.374	0.767	0.867	0.001	0.121	0.130	0.169
		Kazakh *vs.* Tibetan	143	157.216	0.636	0.000	139.297	0.718	0.913	0.854	0.108	0.170	0.185
		Kazakh *vs.* Uyghur	147	156.744	0.638	0.002	145.946	0.685	0.677	0.027	0.470	0.894	0.894
		Kazakh *vs.* Zhuang	139	157.110	0.636	0.000	140.576	0.711	0.404	0.065	0.073	0.073	0.085
		Mongol *vs.* Tibetan	131	154.710	0.646	0.000	134.199	0.745	0.207	0.001	0.636	0.593	0.658
		Mongol *vs.* Uyghur	129	146.807	0.681	0.000	134.102	0.746	0.147	0.000	0.001	0.040	0.048
		Mongol *vs.* Zhuang	132	152.163	0.657	0.000	135.525	0.738	0.177	0.086	0.935	0.322	0.357
		Tibetan *vs.* Uyghur	140	156.914	0.637	0.000	142.859	0.700	0.205	0.010	0.006	0.047	0.103
		Tibetan *vs.* Zhuang	140	161.649	0.619	0.000	142.603	0.701	0.292	0.077	0.551	0.939	1.000
		Uyghur *vs.* Zhuang	143	157.043	0.637	0.000	143.667	0.696	0.462	0.000	0.000	0.009	0.013
	Significant Difference (%)					100%(21/21)			14.3%(3/21)	38.10%	38.10%	38.10%	33.33%
Genus	Rural	Bai *vs.* Han	362	411.319	0.243	0.000	372.486	0.268	0.074	0.664	0.658	0.587	0.403

		Bai *vs.* Kazakh	254	304.283	0.329	0.000	268.927	0.372	0.175	0.410	0.325	0.173	0.173
		Bai *vs.* Mongol	366	426.998	0.234	0.000	386.148	0.259	0.000	0.000	0.000	0.001	0.003
		Bai *vs.* Tibetan	310	362.083	0.276	0.000	324.701	0.308	0.004	0.278	0.004	0.002	0.002
		Bai *vs.* Uyghur	266	305.541	0.327	0.000	273.617	0.365	0.311	0.066	0.133	0.417	0.499
		Bai *vs.* Zhuang	321	366.456	0.273	0.000	330.207	0.303	0.061	0.872	0.484	0.472	0.677
		Han *vs.* Kazakh	275	323.958	0.309	0.000	292.655	0.342	0.120	0.644	0.255	0.069	0.056
		Han *vs.* Mongol	422	455.109	0.220	0.000	415.487	0.241	0.821	0.000	0.000	0.000	0.000
		Han *vs.* Tibetan	332	404.642	0.247	0.000	364.733	0.274	0.000	0.101	0.005	0.007	0.006
		Han *vs.* Uyghur	289	321.832	0.311	0.000	295.026	0.339	0.362	0.074	0.095	0.154	0.218
		Han *vs.* Zhuang	343	406.768	0.246	0.000	359.817	0.278	0.017	0.792	0.642	0.934	0.914
		Kazakh *vs.* Mongol	276	352.740	0.283	0.000	326.318	0.306	0.000	0.005	0.025	0.138	0.255
		Kazakh *vs.* Tibetan	253	284.051	0.352	0.000	251.318	0.398	0.557	0.167	0.020	0.005	0.004
		Kazakh *vs.* Uyghur	238	271.482	0.368	0.000	238.152	0.420	0.512	0.310	0.605	1.000	0.863
		Kazakh *vs.* Zhuang	256	297.654	0.336	0.000	265.805	0.376	0.144	0.518	0.111	0.154	0.142
		Mongol *vs.* Tibetan	343	413.514	0.242	0.000	374.486	0.267	0.000	0.000	0.000	0.000	0.000
		Mongol *vs.* Uyghur	286	352.307	0.284	0.000	328.415	0.304	0.001	0.004	0.171	0.239	0.210
		Mongol *vs.* Zhuang	344	418.644	0.239	0.000	374.411	0.267	0.000	0.000	0.000	0.001	0.002
		Tibetan *vs.* Uyghur	257	284.163	0.352	0.000	253.520	0.394	0.659	0.005	0.003	0.010	0.013
		Tibetan *vs.* Zhuang	298	353.293	0.283	0.000	308.855	0.324	0.018	0.222	0.026	0.017	0.019
		Uyghur *vs.* Zhuang	265	297.347	0.336	0.000	266.307	0.376	0.446	0.057	0.054	0.254	0.392
	Significant Difference (%)					100%(21/21)			42.9%(9/21)	28.57%	52.385	33.335	38.10%
	Urban	Bai *vs.* Han	288	354.247	0.282	0.000	311.346	0.321	0.019	0.509	0.939	0.872	0.993
		Bai *vs.* Kazakh	250	296.549	0.337	0.000	254.319	0.393	0.194	0.274	0.488	0.981	0.943
		Bai *vs.* Mongol	239	278.025	0.360	0.000	240.436	0.416	0.425	0.006	0.713	0.911	0.987
		Bai *vs.* Tibetan	255	311.686	0.321	0.000	264.065	0.379	0.036	0.315	0.621	0.561	0.621
		Bai *vs.* Uyghur	263	307.483	0.325	0.000	278.467	0.359	0.013	0.000	0.015	0.176	0.252
		Bai *vs.* Zhuang	251	293.113	0.341	0.000	260.312	0.384	0.071	0.807	0.761	0.911	0.785
		Han *vs.* Kazakh	308	353.817	0.283	0.000	310.918	0.322	0.424	0.512	0.657	0.790	0.790
		Han *vs.* Mongol	285	369.880	0.270	0.000	332.268	0.301	0.000	0.001	0.478	0.863	0.995
		Han *vs.* Tibetan	316	387.553	0.258	0.000	327.654	0.305	0.159	0.699	0.397	0.536	0.660
		Han *vs.* Uyghur	330	363.494	0.275	0.000	316.380	0.316	0.858	0.001	0.009	0.040	0.081
		Han *vs.* Zhuang	309	371.872	0.269	0.000	336.429	0.297	0.001	0.371	0.883	0.883	0.716
		Kazakh *vs.* Mongol	249	289.425	0.346	0.000	248.966	0.402	0.499	0.001	0.229	0.511	0.674
		Kazakh *vs.* Tibetan	282	324.367	0.308	0.000	281.223	0.356	0.583	0.782	0.440	0.717	0.928
		Kazakh *vs.* Uyghur	285	317.742	0.315	0.000	287.000	0.348	0.368	0.017	0.077	0.347	0.538
		Kazakh *vs.* Zhuang	272	305.698	0.327	0.000	271.430	0.368	0.552	0.161	0.468	0.319	0.371
		Mongol *vs.* Tibetan	253	315.451	0.317	0.000	263.064	0.380	0.071	0.005	0.867	0.867	0.819
		Mongol *vs.* Uyghur	259	299.212	0.334	0.000	266.023	0.376	0.216	0.000	0.002	0.037	0.102
		Mongol *vs.* Zhuang	260	292.324	0.342	0.000	261.455	0.382	0.411	0.076	0.248	0.880	0.569
		Tibetan *vs.* Uyghur	291	333.384	0.300	0.000	296.488	0.337	0.163	0.006	0.021	0.114	0.277
		Tibetan *vs.* Zhuang	272	326.658	0.306	0.000	280.729	0.356	0.091	0.286	0.593	0.772	0.795
		Uyghur *vs.* Zhuang	282	313.318	0.319	0.000	282.555	0.354	0.448	0.001	0.007	0.018	0.031
	Significant Difference (%)					100%(21/21)			23.8%(5/21)	47.62%	23.81%	23.81%	14.29%
Species	Rural	Bai *vs.* Han	639	1029.807	0.097	0.000	901.585	0.111	0.000	0.252	0.174	0.664	0.664
		Bai *vs.* Kazakh	534	681.262	0.147	0.000	604.847	0.165	0.018	0.369	1.000	0.604	0.836
		Bai *vs.* Mongol	640	1057.792	0.095	0.000	946.680	0.106	0.000	0.000	0.000	0.000	0.001
		Bai *vs.* Tibetan	618	842.976	0.119	0.000	749.887	0.133	0.000	0.761	0.057	0.014	0.011
		Bai *vs.* Uyghur	536	683.827	0.146	0.000	613.110	0.163	0.010	0.025	0.084	0.58	1.000

		Bai *vs.* Zhuang	601	879.805	0.114	0.000	761.686	0.131	0.000	0.299	0.7	0.149	0.042
		Han *vs.* Kazakh	690	866.333	0.115	0.000	729.227	0.137	0.191	0.831	0.736	0.807	0.972
		Han *vs.* Mongol	1065	1237.885	0.081	0.000	1079.864	0.093	0.212	0.000	0.000	0.000	0.000
		Han *vs.* Tibetan	864	1093.864	0.091	0.000	937.016	0.107	0.000	0.178	0.015	0.035	0.078
		Han *vs.* Uyghur	714	861.455	0.116	0.000	737.592	0.136	0.303	0.169	0.320	0.600	0.600
		Han *vs.* Zhuang	893	1122.928	0.089	0.000	933.925	0.107	0.020	0.847	0.190	0.157	0.169
		Kazakh *vs.* Mongol	719	922.676	0.108	0.000	849.047	0.118	0.002	0.002	0.002	0.005	0.010
		Kazakh *vs.* Tibetan	656	759.324	0.132	0.000	648.549	0.154	0.587	0.330	0.188	0.144	0.154
		Kazakh *vs.* Uyghur	584	680.638	0.147	0.000	582.316	0.172	0.585	0.340	0.387	0.666	0.666
		Kazakh *vs.* Zhuang	644	776.557	0.129	0.000	665.507	0.150	0.206	0.686	0.706	0.619	0.328
		Mongol *vs.* Tibetan	900	1112.383	0.090	0.000	988.627	0.101	0.000	0.000	0.000	0.000	0.000
		Mongol *vs.* Uyghur	718	923.623	0.108	0.000	855.534	0.117	0.000	0.002	0.015	0.031	0.051
		Mongol *vs.* Zhuang	902	1142.253	0.088	0.000	1003.109	0.100	0.000	0.000	0.000	0.000	0.000
		Tibetan *vs.* Uyghur	654	759.097	0.132	0.000	655.623	0.153	0.437	0.042	0.012	0.100	0.125
		Tibetan *vs.* Zhuang	773	969.253	0.103	0.000	818.825	0.122	0.000	0.304	0.131	0.224	0.338
		Uyghur *vs.* Zhuang	640	779.761	0.128	0.000	666.307	0.150	0.116	0.124	0.094	0.254	0.222
	Significant Difference (%)					100%(21/21)			61.9%(13/21)	42.86%	23.81%	14.29%	4.76%
	Urban	Bai *vs.* Han	601	881.550	0.113	0.000	790.673	0.126	0.000	0.945	0.725	0.725	0.455
		Bai *vs.* Kazakh	552	771.006	0.130	0.000	662.691	0.151	0.000	0.329	0.903	0.542	0.715
		Bai *vs.* Mongol	538	702.195	0.142	0.000	611.347	0.164	0.000	0.007	0.101	0.199	0.306
		Bai *vs.* Tibetan	542	693.550	0.144	0.000	592.133	0.169	0.025	0.033	0.026	0.928	0.964
		Bai *vs.* Uyghur	573	787.446	0.127	0.000	698.116	0.143	0.001	0.008	0.067	0.171	0.394
		Bai *vs.* Zhuang	548	758.216	0.132	0.000	659.710	0.152	0.000	0.313	0.465	0.505	0.548
		Han *vs.* Kazakh	787	932.278	0.107	0.000	787.660	0.127	0.477	0.536	0.750	0.986	0.832
		Han *vs.* Mongol	730	969.619	0.103	0.000	845.011	0.118	0.000	0.000	0.145	0.402	0.527
		Han *vs.* Tibetan	711	966.220	0.103	0.000	818.865	0.122	0.004	0.040	0.340	0.420	0.147
		Han *vs.* Uyghur	853	967.063	0.103	0.000	804.510	0.124	0.940	0.004	0.035	0.126	0.157
		Han *vs.* Zhuang	814	996.728	0.100	0.000	867.050	0.115	0.004	0.176	0.569	0.883	0.924
		Kazakh *vs.* Mongol	632	764.634	0.131	0.000	635.373	0.157	0.392	0.001	0.468	0.578	0.533
		Kazakh *vs.* Tibetan	628	796.213	0.126	0.000	679.734	0.147	0.007	0.281	0.543	0.888	0.963
		Kazakh *vs.* Uyghur	730	836.723	0.120	0.000	729.237	0.137	0.520	0.044	0.068	0.186	0.295
		Kazakh *vs.* Zhuang	680	810.803	0.123	0.000	682.215	0.147	0.426	0.076	0.749	0.853	0.801
		Mongol *vs.* Tibetan	570	755.787	0.132	0.000	632.280	0.158	0.006	0.000	0.001	0.019	0.016
		Mongol *vs.* Uyghur	665	794.974	0.126	0.000	671.461	0.149	0.319	0.000	0.002	0.034	0.074
		Mongol *vs.* Zhuang	650	781.055	0.128	0.000	660.042	0.152	0.116	0.054	0.229	0.333	0.322
		Tibetan *vs.* Uyghur	660	817.899	0.122	0.000	719.258	0.139	0.007	0.123	0.441	0.263	0.941
		Tibetan *vs.* Zhuang	624	804.199	0.124	0.000	685.022	0.146	0.002	0.001	0.008	0.055	0.096
		Uyghur *vs.* Zhuang	726	840.918	0.119	0.000	715.835	0.140	0.651	0.000	0.010	0.127	0.261
	Significant Difference (%)					100%(21/21)			61.9%(13/21)	57.14%	28.57%	9.52%	4.76%

Table S6. The results of shared species analyses (SSA) and Wilcoxon tests for the differences in diversity (Hill numbers) with design **Scheme-2B** (The pair-wise ethnicity comparisons among 7 ethnic groups, with urban & rural lifestyles pooled)

Taxon	Treatment	Observed Shared OTUs	Reads randomization			Samples randomization			P-value of Wilcoxon test for the Hill numbers			
			Expected Shared OTUs	Reduction (%) of shared OTUs (eqn. 3)	*P*-value	Expected Shared OTUs	Reduction (%) of shared OTUs (eqn. 3)	*P*-value	*q*=0	*q*=1	*q*=2	*q*=3
Phylum	Bai *vs*. Han	36	37.032	-2.867	0.321	35.939	0.169	0.602	0.623	0.245	0.105	0.072
	Bai *vs*. Kazakh	28	32.988	-17.814	0.003	30.362	-8.436	0.143	0.278	0.001	0.036	0.068
	Bai *vs*. Mongol	35	38.521	-10.060	0.025	35.181	-0.517	0.568	0.054	0.000	0.005	0.014
	Bai *vs*. Tibetan	31	34.878	-12.510	0.009	31.490	-1.581	0.493	0.078	0.017	0.001	0.001
	Bai *vs*. Uyghur	30	34.557	-15.190	0.000	32.416	-8.053	0.147	0.000	0.000	0.003	0.007
	Bai *vs*. Zhuang	34	35.499	-4.409	0.239	33.757	0.715	0.702	0.769	0.485	0.990	0.854
	Han *vs*. Kazakh	29	34.040	-17.379	0.004	33.027	-13.886	0.025	0.564	0.000	0.000	0.001
	Han *vs*. Mongol	37	38.459	-3.943	0.243	37.321	-0.868	0.535	0.044	0.000	0.000	0.000
	Han *vs*. Tibetan	33	37.144	-12.558	0.002	35.069	-6.270	0.136	0.015	0.000	0.000	0.000
	Han *vs*. Uyghur	32	35.568	-11.150	0.016	33.283	-4.009	0.318	0.000	0.000	0.000	0.000
	Han *vs*. Zhuang	36	36.862	-2.394	0.352	35.492	1.411	0.782	0.344	0.061	0.068	0.071
	Kazakh *vs*. Mongol	30	34.713	-15.710	0.011	32.422	-8.073	0.132	0.327	0.417	0.559	0.720
	Kazakh *vs*. Tibetan	29	31.469	-8.514	0.101	29.451	-1.555	0.517	0.014	0.113	0.929	0.504
	Kazakh *vs*. Uyghur	27	33.695	-24.796	0.000	30.634	-13.459	0.013	0.005	0.448	0.406	0.434
	Kazakh *vs*. Zhuang	29	32.626	-12.503	0.026	30.765	-6.086	0.192	0.210	0.003	0.013	0.020
	Mongol *vs*. Tibetan	35	38.168	-9.051	0.036	35.761	-2.174	0.419	0.000	0.192	0.459	0.081
	Mongol *vs*. Uyghur	29	37.131	-28.038	0.000	33.546	-15.676	0.010	0.054	0.099	0.267	0.418
	Mongol *vs*. Zhuang	34	38.300	-12.647	0.003	35.504	-4.424	0.227	0.011	0.001	0.001	0.001
	Tibetan *vs*. Uyghur	27	33.274	-23.237	0.000	29.427	-8.989	0.093	0.000	0.014	0.600	0.932
	Tibetan *vs*. Zhuang	33	35.739	-8.300	0.038	32.799	0.609	0.689	0.137	0.073	0.001	0.000
	Uyghur *vs*. Zhuang	32	35.111	-9.722	0.046	32.718	-2.244	0.442	0.623	0.245	0.105	0.072
	Significant Difference (%)				76.2%(16/21)			14.3%(3/21)	47.62%	38.10%	38.10%	38.10%
Family	Bai *vs*. Han	212	227.492	-7.308	0.000	212.921	-0.434	0.453	0.337	1.000	0.954	0.939
	Bai *vs*. Kazakh	171	184.298	-7.777	0.000	170.327	0.394	0.550	0.165	0.104	0.367	0.531
	Bai *vs*. Mongol	204	223.874	-9.742	0.000	206.407	-1.180	0.306	0.102	0.003	0.011	0.031
	Bai *vs*. Tibetan	186	205.098	-10.268	0.000	185.797	0.109	0.580	0.945	0.118	0.074	0.062
	Bai *vs*. Uyghur	175	191.390	-9.366	0.000	174.470	0.303	0.571	0.000	0.000	0.012	0.042
	Bai *vs*. Zhuang	189	201.605	-6.669	0.000	187.167	0.970	0.747	0.739	0.650	0.990	0.784
	Han *vs*. Kazakh	183	200.490	-9.557	0.000	188.799	-3.169	0.214	0.482	0.040	0.206	0.431
	Han *vs*. Mongol	228	241.824	-6.063	0.000	226.332	0.732	0.664	0.177	0.001	0.002	0.010
	Han *vs*. Tibetan	195	230.908	-18.414	0.000	211.290	-8.354	0.002	0.251	0.101	0.084	0.049
	Han *vs*. Uyghur	189	206.003	-8.996	0.001	189.982	-0.520	0.479	0.001	0.000	0.004	0.025
	Han *vs*. Zhuang	197	225.047	-14.237	0.000	208.981	-6.082	0.004	0.148	0.667	0.868	0.599
	Kazakh *vs*. Mongol	179	202.492	-13.124	0.000	188.960	-5.564	0.071	0.728	0.271	0.336	0.293
	Kazakh *vs*. Tibetan	161	177.745	-10.401	0.000	163.644	-1.642	0.311	0.163	0.023	0.056	0.087
	Kazakh *vs*. Uyghur	164	176.271	-7.482	0.000	164.028	-0.017	0.562	0.035	0.157	0.283	0.392
	Kazakh *vs*. Zhuang	162	179.038	-10.517	0.000	167.760	-3.556	0.126	0.068	0.069	0.248	0.379
	Mongol *vs*. Tibetan	194	224.767	-15.859	0.000	207.003	-6.703	0.004	0.032	0.000	0.000	0.001
	Mongol *vs*. Uyghur	180	209.385	-16.325	0.000	192.530	-6.961	0.039	0.341	0.565	0.954	0.943
	Mongol *vs*. Zhuang	188	220.950	-17.527	0.000	203.176	-8.072	0.000	0.036	0.004	0.004	0.008
	Tibetan *vs*. Uyghur	165	184.044	-11.542	0.000	167.948	-1.787	0.299	0.000	0.000	0.001	0.002
	Tibetan *vs*. Zhuang	176	202.086	-14.822	0.000	182.881	-3.910	0.030	0.828	0.066	0.111	0.111

	Uyghur *vs.* Zhuang	168	186.654	-11.104	0.000	172.125	-2.455	0.218	0.000	0.000	0.004	0.020
	Significant Difference (%)				100%(21/21)			28.6%(6/21)	33.33%	47.62%	38.10%	42.86%
Genus	Bai *vs.* Han	425	478.339	-12.550	0.000	434.291	-2.186	0.143	0.524	0.735	0.558	0.493
	Bai *vs.* Kazakh	337	379.636	-12.652	0.000	338.627	-0.483	0.420	0.215	0.304	0.428	0.487
	Bai *vs.* Mongol	412	461.401	-11.991	0.000	419.248	-1.759	0.144	0.084	0.043	0.056	0.068
	Bai *vs.* Tibetan	367	423.277	-15.334	0.000	375.945	-2.437	0.063	0.682	0.009	0.005	0.008
	Bai *vs.* Uyghur	352	393.802	-11.876	0.000	352.212	-0.060	0.488	0.000	0.008	0.103	0.162
	Bai *vs.* Zhuang	368	414.566	-12.654	0.000	376.728	-2.372	0.068	0.908	0.644	0.464	0.474
	Han *vs.* Kazakh	368	417.582	-13.473	0.000	378.674	-2.901	0.208	0.402	0.253	0.150	0.138
	Han *vs.* Mongol	476	510.704	-7.291	0.000	468.525	1.570	0.820	0.109	0.012	0.005	0.005
	Han *vs.* Tibetan	402	484.360	-20.488	0.000	433.673	-7.879	0.000	0.237	0.004	0.009	0.009
	Han *vs.* Uyghur	392	424.834	-8.376	0.000	382.578	2.404	0.756	0.000	0.003	0.016	0.038
	Han *vs.* Zhuang	397	475.556	-19.787	0.000	428.702	-7.985	0.000	0.429	0.814	0.868	0.776
	Kazakh *vs.* Mongol	363	414.451	-14.174	0.000	375.582	-3.466	0.150	0.728	0.362	0.362	0.396
	Kazakh *vs.* Tibetan	329	374.248	-13.753	0.000	333.738	-1.440	0.275	0.106	0.014	0.012	0.013
	Kazakh *vs.* Uyghur	330	361.393	-9.513	0.000	329.212	0.239	0.585	0.015	0.087	0.477	0.745
	Kazakh *vs.* Zhuang	325	362.392	-11.505	0.000	328.195	-0.983	0.342	0.161	0.112	0.106	0.112
	Mongol *vs.* Tibetan	387	458.724	-18.533	0.000	408.458	-5.545	0.001	0.023	0.000	0.000	0.000
	Mongol *vs.* Uyghur	383	431.928	-12.775	0.000	388.463	-1.426	0.310	0.309	0.591	0.985	0.781
	Mongol *vs.* Zhuang	383	456.838	-19.279	0.000	408.981	-6.784	0.000	0.039	0.020	0.009	0.010
	Tibetan *vs.* Uyghur	340	380.712	-11.974	0.000	342.225	-0.654	0.415	0.000	0.000	0.000	0.001
	Tibetan *vs.* Zhuang	351	414.881	-18.200	0.000	366.199	-4.330	0.002	0.767	0.018	0.015	0.022
	Uyghur *vs.* Zhuang	342	378.152	-10.571	0.000	340.663	0.391	0.566	0.000	0.001	0.013	0.039
	Significant Difference (%)				100%(21/21)			23.8%(5/21)	33.33%	57.14%	47.62%	47.62%
Species	Bai *vs.* Han	744	1208.054	-62.373	0.000	1076.670	-44.714	0.000	0.360	0.171	0.841	0.393
	Bai *vs.* Kazakh	665	879.433	-32.246	0.000	762.760	-14.701	0.000	0.146	0.862	0.917	0.884
	Bai *vs.* Mongol	731	1130.470	-54.647	0.000	1008.561	-37.970	0.000	0.054	0.011	0.047	0.135
	Bai *vs.* Tibetan	700	925.634	-32.233	0.000	820.201	-17.172	0.000	0.277	0.632	0.087	0.091
	Bai *vs.* Uyghur	684	906.586	-32.542	0.000	798.891	-16.797	0.000	0.000	0.005	0.099	0.358
	Bai *vs.* Zhuang	709	999.428	-40.963	0.000	876.365	-23.606	0.000	0.912	0.535	0.216	0.082
	Han *vs.* Kazakh	958	1133.593	-18.329	0.000	985.145	-2.834	0.208	0.493	0.589	0.939	0.775
	Han *vs.* Mongol	1242	1401.567	-12.848	0.000	1245.442	-0.277	0.405	0.129	0.018	0.021	0.017
	Han *vs.* Tibetan	996	1290.312	-29.549	0.000	1124.290	-12.881	0.000	0.843	0.100	0.138	0.375
	Han *vs.* Uyghur	1006	1155.506	-14.861	0.000	989.748	1.616	0.665	0.001	0.024	0.119	0.140
	Han *vs.* Zhuang	1086	1327.745	-22.260	0.000	1142.233	-5.178	0.002	0.305	0.181	0.230	0.328
	Kazakh *vs.* Mongol	948	1104.585	-16.517	0.000	989.524	-4.380	0.094	0.600	0.053	0.062	0.116
	Kazakh *vs.* Tibetan	837	962.654	-15.012	0.000	853.409	-1.960	0.218	0.728	0.537	0.409	0.276
	Kazakh *vs.* Uyghur	854	966.108	-13.127	0.000	850.084	0.459	0.675	0.032	0.042	0.157	0.251
	Kazakh *vs.* Zhuang	871	1002.419	-15.088	0.000	866.567	0.509	0.572	0.177	0.691	0.607	0.438
	Mongol *vs.* Tibetan	982	1203.289	-22.535	0.000	1075.437	-9.515	0.000	0.159	0.003	0.002	0.005
	Mongol *vs.* Uyghur	997	1139.007	-14.243	0.000	1011.849	-1.489	0.309	0.404	0.964	0.674	0.674
	Mongol *vs.* Zhuang	1039	1250.254	-20.332	0.000	1102.781	-6.139	0.000	0.057	0.005	0.003	0.004
	Tibetan *vs.* Uyghur	861	978.113	-13.602	0.000	870.891	-1.149	0.303	0.005	0.006	0.018	0.057
	Tibetan *vs.* Zhuang	900	1101.790	-22.421	0.000	963.033	-7.004	0.000	0.242	0.918	0.860	0.905
	Uyghur *vs.* Zhuang	901	1030.257	-14.346	0.000	892.641	0.928	0.646	0.000	0.002	0.028	0.052
	Significant Difference (%)				100%(21/21)			52.4%(11/21)	23.81%	42.86%	28.57%	19.05%

Table S7. The *mean* and *standard error* of the Hill numbers (diversity) for each ethnicity with both lifestyles pooled with design **Scheme-2B**.

Dataset	Treatments	Statistics	*q*=0	*q*=1	*q*=2	*q*=3
Phylum	Bai	Mean	16.140	2.541	1.837	1.671
		Std. Err.	0.440	0.102	0.076	0.065
	Han	Mean	16.198	2.410	1.703	1.546
		Std. Err.	0.301	0.064	0.047	0.041
	Kazakh	Mean	16.545	3.208	2.176	1.910
		Std. Err.	0.513	0.178	0.132	0.108
	Mongol	Mean	17.417	3.043	2.067	1.828
		Std. Err.	0.448	0.093	0.057	0.047
	Tibetan	Mean	14.977	2.923	2.205	2.019
		Std. Err.	0.387	0.120	0.093	0.082
	Uyghur	Mean	18.952	3.501	2.422	2.136
		Std. Err.	0.558	0.211	0.182	0.163
	Zhuang	Mean	15.696	2.626	1.834	1.654
		Std. Err.	0.357	0.106	0.084	0.073
	Total samples	Mean	16.561	2.893	2.035	1.823
		Std. Err.	0.429	0.125	0.096	0.083
Family	Bai	Mean	74.023	12.522	8.135	6.866
		Std. Err.	2.509	0.454	0.400	0.375
	Han	Mean	75.989	12.380	8.015	6.792
		Std. Err.	1.632	0.355	0.279	0.251
	Kazakh	Mean	78.455	13.702	8.822	7.365
		Std. Err.	3.225	0.920	0.796	0.727
	Mongol	Mean	83.667	15.416	10.043	8.388
		Std. Err.	3.260	0.699	0.531	0.478
	Tibetan	Mean	71.814	11.474	7.140	5.904
		Std. Err.	2.095	0.576	0.464	0.409
	Uyghur	Mean	86.952	15.879	10.143	8.384
		Std. Err.	2.720	0.675	0.609	0.578
	Zhuang	Mean	71.848	12.633	8.019	6.664
		Std. Err.	1.796	0.430	0.350	0.316
	Total samples	Mean	77.535	13.429	8.617	7.195
		Std. Err.	2.462	0.587	0.490	0.448
Genus	Bai	Mean	136.628	21.236	12.235	9.787
		Std. Err.	4.871	0.872	0.612	0.528
	Han	Mean	140.110	20.841	11.735	9.339
		Std. Err.	3.313	0.629	0.417	0.347
	Kazakh	Mean	146.591	22.072	12.945	10.467
		Std. Err.	6.854	1.404	1.032	0.892
	Mongol	Mean	158.667	24.892	14.393	11.502
		Std. Err.	6.964	1.211	0.770	0.636
	Tibetan	Mean	130.186	17.243	9.433	7.515
		Std. Err.	4.469	1.073	0.748	0.630
	Uyghur	Mean	165.333	25.732	14.429	11.294
		Std. Err.	5.177	1.306	0.945	0.804
	Zhuang	Mean	134.283	20.535	11.680	9.300
		Std. Err.	3.855	0.751	0.528	0.454
	Total samples	Mean	144.542	21.793	12.407	9.886
		Std. Err.	5.072	1.035	0.722	0.613

Species	Bai	Mean	305.605	51.361	23.766	17.613
		Std. Err.	9.560	2.474	1.368	1.011
	Han	Mean	326.912	55.378	24.578	16.962
		Std. Err.	8.599	2.342	1.315	0.949
	Kazakh	Mean	338.500	52.940	24.176	17.597
		Std. Err.	18.148	4.766	2.598	1.911
	Mongol	Mean	376.083	70.095	31.923	22.171
		Std. Err.	18.858	4.314	2.225	1.577
	Tibetan	Mean	319.953	48.760	21.374	15.233
		Std. Err.	11.709	3.343	1.759	1.259
	Uyghur	Mean	385.333	68.340	30.317	20.950
		Std. Err.	14.512	4.678	2.991	2.206
	Zhuang	Mean	310.217	50.442	21.838	15.149
		Std. Err.	10.041	2.284	1.204	0.861
	Total samples	Mean	337.515	56.760	25.425	17.954
		Std. Err.	13.061	3.457	1.923	1.396

Table S8. The unique phyla of and shared phyla between rural and urban lifestyle for each ethnic group, *i.e.*, comparing the Rural *vs*. Urban lifestyles of same ethnic group

Ethnic Group	Unique Phylum		Shared Phyla	Ethnic Group	Unique Phylum		Shared Phyla
	Rural	Urban			Rural	Urban	
**Bai**	*Phaeophyceae*	*Deferribacteres*	*Firmicutes*	**Han**	*Cnidaria*	*Crenarchaeota*	*Firmicutes*
	Pinguiophyceae	*Acidobacteria*	*Bacteroidetes*		*Thermodesulfobacteria*	*Acidobacteria*	*Bacteroidetes*
	Ascomycota	*Thermodesulfobacteria*	*Proteobacteria*		*Microsporidia*	*unclassified..derived.from.Archaea.*	*unclassified..derived.from.Bacteria.*
	Aquificae	*Chlorobi*	*Arthropoda*			*Aquificae*	*Proteobacteria*
	Bacillariophyta	*Dictyoglomi*	*unclassified..derived.from.Bacteria.*				*Actinobacteria*
	Cnidaria	*Chrysiogenetes*	*Actinobacteria*				*Arthropoda*
	Basidiomycota		*Euryarchaeota*				*Streptophyta*
	Fibrobacteres		*Streptophyta*				*Euryarchaeota*
	Euglenida		*Fusobacteria*				*Fusobacteria*
			unclassified..derived.from.unclassified.sequences.				*Verrucomicrobia*
			Verrucomicrobia				*unclassified..derived.from.unclassified.sequences.*
			Tenericutes				*Cyanobacteria*
			Synergistetes				*Tenericutes*
			unclassified..derived.from.Viruses.				*Synergistetes*
			Cyanobacteria				*Chordata*
			Chordata				*unclassified..derived.from.Viruses.*
			unclassified..derived.from.Eukaryota.				*unclassified..derived.from.Eukaryota.*
			Spirochaetes				*Spirochaetes*
			Deinococcus.Thermus				*Phaeophyceae*
			Chlorophyta				*Chlorophyta*
			Planctomycetes				*Planctomycetes*
			Apicomplexa				*Deinococcus.Thermus*
			Chloroflexi				*Nitrospirae*
			Nitrospirae				*Deferribacteres*
			Lentisphaerae				*Basidiomycota*
**Kazakh**	*Nitrospirae*	*Microsporidia*	*Firmicutes*				*Apicomplexa*
	Thermodesulfobacteria	*Planctomycetes*	*Bacteroidetes*				*Ascomycota*
		Nematoda	*Euryarchaeota*				*Chloroflexi*
		Apicomplexa	*Actinobacteria*				*Echinodermata*
		Elusimicrobia	*Proteobacteria*				*Pinguiophyceae*
			unclassified..derived.from.Bacteria.				*Fibrobacteres*
			Arthropoda				*Thermotogae*
			Streptophyta				*Chlorobi*
			Fusobacteria				*Bacillariophyta*
			unclassified..derived.from.unclassified.sequences.	**Mongol**	*Phaeophyceae*	*Thaumarchaeota*	*Firmicutes*
			Verrucomicrobia		*Nitrospirae*	*Chlorobi*	*Bacteroidetes*
			Cyanobacteria		*Pinguiophyceae*		*Actinobacteria*
			Synergistetes		*Nematoda*		*Proteobacteria*
			Chordata		*Apicomplexa*		*unclassified..derived.from.Bacteria.*

			Crenarchaeota		Echinodermata		Arthropoda
			unclassified..derived.from.Viruses.		Thermotogae		Euryarchaeota
			Tenericutes		Aquificae		Streptophyta
			Deferribacteres		Dictyoglomi		Fusobacteria
			Deinococcus.Thermus				Verrucomicrobia
			unclassified..derived.from.Eukaryota.				unclassified..derived.from.unclassified.sequences.
			Chlorophyta				Synergistetes
			Dictyoglomi				Cyanobacteria
			Phaeophyceae				Tenericutes
			Spirochaetes				unclassified..derived.from.Viruses.
			Basidiomycota				Chordata
**Tibetan**	Microsporidia	Deferribacteres	Firmicutes				unclassified..derived.from.Eukaryota.
	Basidiomycota	Elusimicrobia	Bacteroidetes				Spirochaetes
	Apicomplexa	Phaeophyceae	unclassified..derived.from.Bacteria.				Chlorophyta
	Planctomycetes	Chloroflexi	Actinobacteria				Deinococcus.Thermus
	Pinguiophyceae	Dictyoglomi	Arthropoda				Basidiomycota
	Crenarchaeota	Chlorobi	Verrucomicrobia				Crenarchaeota
	Fibrobacteres		Euryarchaeota				Planctomycetes
	Thaumarchaeota		Proteobacteria				Microsporidia
			Streptophyta				Ascomycota
			Fusobacteria				Chloroflexi
			unclassified..derived.from.unclassified.sequences.				Fibrobacteres
			unclassified..derived.from.Viruses.				Cnidaria
			Spirochaetes				Deferribacteres
			Cyanobacteria				Lentisphaerae
			Tenericutes	**Uyghur**	Acidobacteria	Phaeophyceae	Firmicutes
			unclassified..derived.from.Eukaryota.		Chlamydiae	Thermotogae	Bacteroidetes
			Chordata		Cnidaria	Bacillariophyta	Proteobacteria
			Synergistetes			Gemmatimonadetes	Euryarchaeota
			Deinococcus.Thermus				unclassified..derived.from.Bacteria.
			Chlorophyta				Actinobacteria
			Thermotogae				Streptophyta
			Ascomycota				Fusobacteria
**Zhuang**	Planctomycetes	Thermodesulfobacteria	Firmicutes				Arthropoda
	Chlorobi	Acidobacteria	Bacteroidetes				unclassified..derived.from.unclassified.sequences.
	unclassified..derived.from.other.sequences.	Fibrobacteres	unclassified..derived.from.Bacteria.				Verrucomicrobia
	Cnidaria	Chlamydiae	Proteobacteria				Synergistetes
	Thermotogae	Elusimicrobia	Arthropoda				Chordata
			Actinobacteria				Spirochaetes
			unclassified..derived.from.unclassified.sequences.				Cyanobacteria
			Euryarchaeota				unclassified..derived.from.Viruses.
			Fusobacteria				Tenericutes
			Streptophyta				unclassified..derived.from.Eukaryota.

			Verrucomicrobia				*Chlorophyta*
			unclassified..derived .from.Viruses.				*Elusimicrobia*
			Tenericutes				*Deinococcus.Thermus*
			Synergistetes				*Ascomycota*
			Cyanobacteria				*Planctomycetes*
			Spirochaetes				*Basidiomycota*
			Chordata				*Echinodermata*
			unclassified..derived .from.Eukaryota.				*Deferribacteres*
			Pinguiophyceae				*Nitrospirae*
			Deinococcus.Therm us				*Thermodesulfobacteria*
			Chlorophyta				
			Basidiomycota				
			Ascomycota				
			Crenarchaeota				
			Apicomplexa				
			Phaeophyceae				
			Deferribacteres				
			Chloroflexi				
			Nitrospirae				

Table S9. The means of the *Normalized Stochastic Ratio* (*NSR*) of the Chinese gut microbiome with **Scheme-1A** (comparing the Rural *vs*. Urban lifestyles of same ethnicity), and the percentage with significant differences from Wilcoxon tests for the differences in the *NSR* between rural and urban groups (*P*-value=0.05)

Group	Treatment	Rural	Urban	Wilcoxon test for the difference in NSR		
		NSR (Normalized Stochasticity Ratio)	NSR (Normalized Stochasticity Ratio)	≠	Rural > Urban	Rural < Urban
OTU-table-Phylum	Bai	0.622	0.584	0.377	0.188	0.812
	Han	0.570	0.591	0.810	0.405	0.595
	Kazakh	0.616	0.343	0.002	0.001	0.999
	Mongol	0.668	0.478	0.000	0.000	1.000
	Tibetan	0.385	0.341	0.432	0.216	0.784
	Uyghur	0.344	0.576	0.005	0.998	0.002
	Zhuang	0.503	0.536	0.868	0.434	0.566
Mean (Std Error) | or Percentage (%) with Significant Difference		0.530 (0.047)	0.493 (0.042)	42.9%(3/7)	28.6%(2/7)	14.3%(1/7)
OTU-table-Family	Bai	0.372	0.393	0.631	0.685	0.315
	Han	0.320	0.319	0.599	0.300	0.700
	Kazakh	0.331	0.179	0.063	0.031	0.969
	Mongol	0.501	0.263	0.000	0.000	1.000
	Tibetan	0.215	0.212	0.509	0.254	0.746
	Uyghur	0.243	0.357	0.146	0.928	0.073
	Zhuang	0.296	0.261	0.521	0.260	0.740
Mean (Std Error) | or Percentage (%) with Significant Difference		0.325 (0.035)	0.283 (0.029)	14.3%(1/7)	28.6%(2/7)	0(0/7)
OTU-table-Genus	Bai	0.312	0.341	0.497	0.752	0.249
	Han	0.264	0.267	0.921	0.539	0.461
	Kazakh	0.281	0.153	0.095	0.048	0.953
	Mongol	0.455	0.227	0.000	0.000	1.000
	Tibetan	0.192	0.205	0.709	0.354	0.646
	Uyghur	0.219	0.315	0.224	0.889	0.112
	Zhuang	0.247	0.227	0.906	0.547	0.453
Mean (Std Error) | or Percentage (%) with Significant Difference		0.281 (0.033)	0.248 (0.025)	14.3%(1/7)	28.6%(2/7)	0(0/7)
OTU-table-Species	Bai	0.295	0.286	0.898	0.552	0.449
	Han	0.215	0.232	0.072	0.964	0.036
	Kazakh	0.243	0.131	0.065	0.033	0.968
	Mongol	0.368	0.195	0.000	0.000	1.000
	Tibetan	0.151	0.168	0.453	0.227	0.774
	Uyghur	0.175	0.260	0.415	0.795	0.207
	Zhuang	0.202	0.193	0.406	0.797	0.203
Mean (Std Error) | or Percentage (%) with Significant Difference		0.236 (0.028)	0.209 (0.020)	14.3%(1/7)	28.6%(2/7)	14.3%(1/7)

Table S10. The means of the *Normalized Stochastic Ratio* (*NSR*) of the Chinese gut microbiome under design **Scheme-1B** (Comparing the Rural *vs*. Urban lifestyles, with all 7 ethnic groups combined for each lifestyle), and the percentage with significant difference from Wilcoxon test for the differences between rural and urban groups in *NSR* (*P*-value=0.05)

**Group**	**Rural**	**Urban**	**Wilcoxon test for the NSR**		
	**Normalized Stochasticity Ratio (NSR)**	**Normalized Stochasticity Ratio (NSR)**	≠	>	<
OTU-table-Phylum	0.508	0.506	0.006	0.003	0.997
OTU-table-Family	0.299	0.276	0.000	0.000	1.000
OTU-table-Genus	0.254	0.235	0.002	0.001	0.999
OTU-table-Species	0.204	0.198	0.765	0.618	0.382
Mean (Std Error) | or Percentage (%) with Significant Difference	0.316 (0.058)	0.304 (0.060)	75%(3/4)	75%(3/4)	0(0/4)

Table S11. The means of the *Normalized Stochastic Ratio* (*NSR*) of the Chinese gut microbiomes under design **Scheme-2A** (The pair-wise comparison of ethnic groups for rural and urban life styles, respectively, at four taxon levels), and the percentage of ethnic pairs with significant differences in the NSR from the Wilcoxon (*P*-value=0.05)

Group	Treatment	Ethnicity1	Ethnicity2	Wilcoxon test for the NSR		
		Normalized Stochasticity Ratio (NSR)	Normalized Stochasticity Ratio (NSR)	≠	>	<
OTU-table-Phylum	**Rural lifestyles**					
	Bai *vs*. Han	0.622	0.570	0.046	0.023	0.977
	Bai *vs*. Kazakh	0.622	0.616	0.539	0.269	0.731
	Bai *vs*. Mongol	0.622	0.668	0.511	0.745	0.255
	Bai *vs*. Tibetan	0.622	0.385	0.000	0.000	1.000
	Bai *vs*. Uyghur	0.622	0.344	0.000	0.000	1.000
	Bai *vs*. Zhuang	0.622	0.503	0.000	0.000	1.000
	Han *vs*. Kazakh	0.570	0.616	0.852	0.574	0.426
	Han *vs*. Mongol	0.570	0.668	0.002	0.999	0.001
	Han *vs*. Tibetan	0.570	0.385	0.000	0.000	1.000
	Han *vs*. Uyghur	0.570	0.344	0.000	0.000	1.000
	Han *vs*. Zhuang	0.570	0.503	0.011	0.006	0.994
	Kazakh *vs*. Mongol	0.616	0.668	0.278	0.861	0.139
	Kazakh *vs*. Tibetan	0.616	0.385	0.000	0.000	1.000
	Kazakh *vs*. Uyghur	0.616	0.344	0.006	0.003	0.997
	Kazakh *vs*. Zhuang	0.616	0.503	0.184	0.092	0.908
	Mongol *vs*. Tibetan	0.668	0.385	0.000	0.000	1.000
	Mongol *vs*. Uyghur	0.668	0.344	0.000	0.000	1.000
	Mongol *vs*. Zhuang	0.668	0.503	0.000	0.000	1.000
	Tibetan *vs*. Uyghur	0.385	0.344	0.510	0.255	0.745
	Tibetan *vs*. Zhuang	0.385	0.503	0.000	1.000	0.000
	Uyghur *vs*. Zhuang	0.344	0.503	0.020	0.990	0.010
	Mean (Std Error) | or Percentage (%) with Significant Difference	0.579 (0.020)	0.480 (0.026)	71.4%(15/21)	57.1%(12/21)	14.3%(3/21)
	**Urban lifestyles**					
	Bai *vs*. Han	0.584	0.591	0.565	0.283	0.717
	Bai *vs*. Kazakh	0.584	0.343	0.000	0.000	1.000
	Bai *vs*. Mongol	0.584	0.478	0.001	0.000	1.000
	Bai *vs*. Tibetan	0.584	0.341	0.001	0.000	1.000
	Bai *vs*. Uyghur	0.584	0.576	0.284	0.142	0.859
	Bai *vs*. Zhuang	0.584	0.536	0.019	0.009	0.991
	Han *vs*. Kazakh	0.591	0.343	0.000	0.000	1.000
	Han *vs*. Mongol	0.591	0.478	0.000	0.000	1.000
	Han *vs*. Tibetan	0.591	0.341	0.000	0.000	1.000
	Han *vs*. Uyghur	0.591	0.576	0.516	0.258	0.742
	Han *vs*. Zhuang	0.591	0.536	0.003	0.001	0.999
	Kazakh *vs*. Mongol	0.343	0.478	0.012	0.994	0.006
	Kazakh *vs*. Tibetan	0.343	0.341	1.000	0.501	0.501
	Kazakh *vs*. Uyghur	0.343	0.576	0.001	1.000	0.000
	Kazakh *vs*. Zhuang	0.343	0.536	0.000	1.000	0.000
	Mongol *vs*. Tibetan	0.478	0.341	0.013	0.006	0.994
	Mongol *vs*. Uyghur	0.478	0.576	0.020	0.990	0.010

	Mongol *vs*. Zhuang	0.478	0.536	0.065	0.968	0.032
	Tibetan *vs*. Uyghur	0.341	0.576	0.003	0.999	0.001
	Tibetan *vs*. Zhuang	0.341	0.536	0.001	1.000	0.000
	Uyghur *vs*. Zhuang	0.576	0.536	0.255	0.127	0.873
	Mean (Std Error) | or Percentage (%) with Significant Difference	0.501 (0.024)	0.484 (0.021)	71.4%(15/21)	42.9%(9/21)	33.3%(7/21)
OTU-table-Family	**Rural lifestyles**					
	Bai *vs*. Han	0.372	0.320	0.064	0.032	0.968
	Bai *vs*. Kazakh	0.372	0.331	0.468	0.234	0.766
	Bai *vs*. Mongol	0.372	0.501	0.000	1.000	0.000
	Bai *vs*. Tibetan	0.372	0.215	0.000	0.000	1.000
	Bai *vs*. Uyghur	0.372	0.243	0.053	0.027	0.973
	Bai *vs*. Zhuang	0.372	0.296	0.027	0.014	0.986
	Han *vs*. Kazakh	0.320	0.331	0.995	0.503	0.497
	Han *vs*. Mongol	0.320	0.501	0.000	1.000	0.000
	Han *vs*. Tibetan	0.320	0.215	0.000	0.000	1.000
	Han *vs*. Uyghur	0.320	0.243	0.242	0.121	0.879
	Han *vs*. Zhuang	0.320	0.296	0.368	0.184	0.816
	Kazakh *vs*. Mongol	0.331	0.501	0.011	0.995	0.005
	Kazakh *vs*. Tibetan	0.331	0.215	0.091	0.045	0.955
	Kazakh *vs*. Uyghur	0.331	0.243	0.326	0.163	0.840
	Kazakh *vs*. Zhuang	0.331	0.296	0.632	0.316	0.684
	Mongol *vs*. Tibetan	0.501	0.215	0.000	0.000	1.000
	Mongol *vs*. Uyghur	0.501	0.243	0.000	0.000	1.000
	Mongol *vs*. Zhuang	0.501	0.296	0.000	0.000	1.000
	Tibetan *vs*. Uyghur	0.215	0.243	0.617	0.692	0.308
	Tibetan *vs*. Zhuang	0.215	0.296	0.037	0.981	0.019
	Uyghur *vs*. Zhuang	0.243	0.296	0.605	0.698	0.302
	Mean (Std Error) | or Percentage (%) with Significant Difference	0.349 (0.017)	0.302 (0.020)	47.6%(10/21)	42.9%(9/21)	19.0%(4/21)
	**Urban lifestyles**					
	Bai *vs*. Han	0.393	0.319	0.055	0.028	0.972
	Bai *vs*. Kazakh	0.393	0.179	0.002	0.001	0.999
	Bai *vs*. Mongol	0.393	0.263	0.004	0.002	0.998
	Bai *vs*. Tibetan	0.393	0.212	0.012	0.006	0.994
	Bai *vs*. Uyghur	0.393	0.357	0.469	0.235	0.766
	Bai *vs*. Zhuang	0.393	0.261	0.003	0.002	0.998
	Han *vs*. Kazakh	0.319	0.179	0.002	0.001	0.999
	Han *vs*. Mongol	0.319	0.263	0.059	0.029	0.971
	Han *vs*. Tibetan	0.319	0.212	0.007	0.003	0.997
	Han *vs*. Uyghur	0.319	0.357	0.385	0.807	0.193
	Han *vs*. Zhuang	0.319	0.261	0.049	0.024	0.976
	Kazakh *vs*. Mongol	0.179	0.263	0.038	0.981	0.019
	Kazakh *vs*. Tibetan	0.179	0.212	0.938	0.532	0.469
	Kazakh *vs*. Uyghur	0.179	0.357	0.007	0.996	0.004
	Kazakh *vs*. Zhuang	0.179	0.261	0.055	0.973	0.027
	Mongol *vs*. Tibetan	0.263	0.212	0.066	0.033	0.967
	Mongol *vs*. Uyghur	0.263	0.357	0.058	0.971	0.029
	Mongol *vs*. Zhuang	0.263	0.261	0.991	0.505	0.495
	Tibetan *vs*. Uyghur	0.212	0.357	0.019	0.990	0.010
	Tibetan *vs*. Zhuang	0.212	0.261	0.116	0.942	0.058

	Uyghur *vs*. Zhuang	0.357	0.261	0.069	0.034	0.966
	Mean (Std Error) | or Percentage (%) with Significant Difference	0.297 (0.018)	0.270 (0.013)	47.6%(10/21)	52.4%(11/21)	23.8%(5/21)
OTU-table-Genus	**Rural lifestyles**					
	Bai *vs*. Han	0.312	0.264	0.067	0.033	0.967
	Bai *vs*. Kazakh	0.312	0.281	0.565	0.283	0.718
	Bai *vs*. Mongol	0.312	0.455	0.000	1.000	0.000
	Bai *vs*. Tibetan	0.312	0.192	0.000	0.000	1.000
	Bai *vs*. Uyghur	0.312	0.219	0.139	0.070	0.930
	Bai *vs*. Zhuang	0.312	0.247	0.055	0.028	0.972
	Han *vs*. Kazakh	0.264	0.281	0.836	0.582	0.418
	Han *vs*. Mongol	0.264	0.455	0.000	1.000	0.000
	Han *vs*. Tibetan	0.264	0.192	0.006	0.003	0.997
	Han *vs*. Uyghur	0.264	0.219	0.567	0.284	0.717
	Han *vs*. Zhuang	0.264	0.247	0.500	0.250	0.750
	Kazakh *vs*. Mongol	0.281	0.455	0.010	0.995	0.005
	Kazakh *vs*. Tibetan	0.281	0.192	0.168	0.084	0.916
	Kazakh *vs*. Uyghur	0.281	0.219	0.484	0.242	0.762
	Kazakh *vs*. Zhuang	0.281	0.247	0.524	0.262	0.739
	Mongol *vs*. Tibetan	0.455	0.192	0.000	0.000	1.000
	Mongol *vs*. Uyghur	0.455	0.219	0.000	0.000	1.000
	Mongol *vs*. Zhuang	0.455	0.247	0.000	0.000	1.000
	Tibetan *vs*. Uyghur	0.192	0.219	0.531	0.735	0.266
	Tibetan *vs*. Zhuang	0.192	0.247	0.300	0.850	0.150
	Uyghur *vs*. Zhuang	0.219	0.247	0.995	0.504	0.497
	Mean (Std Error) | or Percentage (%) with Significant Difference	0.301 (0.016)	0.263 (0.018)	38.1%(8/21)	33.3%(7/21)	14.3%(3/21)
	**Urban lifestyles**					
	Bai *vs*. Han	0.341	0.267	0.054	0.027	0.973
	Bai *vs*. Kazakh	0.341	0.153	0.005	0.003	0.997
	Bai *vs*. Mongol	0.341	0.227	0.010	0.005	0.995
	Bai *vs*. Tibetan	0.341	0.205	0.049	0.025	0.976
	Bai *vs*. Uyghur	0.341	0.315	0.650	0.325	0.676
	Bai *vs*. Zhuang	0.341	0.227	0.011	0.005	0.995
	Han *vs*. Kazakh	0.267	0.153	0.007	0.004	0.996
	Han *vs*. Mongol	0.267	0.227	0.214	0.107	0.893
	Han *vs*. Tibetan	0.267	0.205	0.054	0.027	0.973
	Han *vs*. Uyghur	0.267	0.315	0.285	0.857	0.143
	Han *vs*. Zhuang	0.267	0.227	0.187	0.093	0.907
	Kazakh *vs*. Mongol	0.153	0.227	0.034	0.983	0.017
	Kazakh *vs*. Tibetan	0.153	0.205	0.683	0.659	0.342
	Kazakh *vs*. Uyghur	0.153	0.315	0.010	0.995	0.005
	Kazakh *vs*. Zhuang	0.153	0.227	0.056	0.972	0.028
	Mongol *vs*. Tibetan	0.227	0.205	0.158	0.079	0.921
	Mongol *vs*. Uyghur	0.227	0.315	0.084	0.958	0.042
	Mongol *vs*. Zhuang	0.227	0.227	0.967	0.483	0.517
	Tibetan *vs*. Uyghur	0.205	0.315	0.044	0.978	0.022
	Tibetan *vs*. Zhuang	0.205	0.227	0.256	0.872	0.128
	Uyghur *vs*. Zhuang	0.315	0.227	0.102	0.051	0.949
	Mean (Std Error) | or Percentage (%) with Significant Difference	0.245 (0.015)	0.244 (0.010)	38.1%(8/21)	33.3%(7/21)	23.8%(5/21)

OTU-table-Species	**Rural lifestyles**					
	Bai *vs*. Han	0.295	0.215	0.000	0.000	1.000
	Bai *vs*. Kazakh	0.295	0.243	0.188	0.094	0.906
	Bai *vs*. Mongol	0.295	0.368	0.024	0.988	0.012
	Bai *vs*. Tibetan	0.295	0.151	0.000	0.000	1.000
	Bai *vs*. Uyghur	0.295	0.175	0.020	0.010	0.990
	Bai *vs*. Zhuang	0.295	0.202	0.001	0.000	1.000
	Han *vs*. Kazakh	0.215	0.243	0.541	0.730	0.270
	Han *vs*. Mongol	0.215	0.368	0.000	1.000	0.000
	Han *vs*. Tibetan	0.215	0.151	0.046	0.023	0.977
	Han *vs*. Uyghur	0.215	0.175	0.834	0.417	0.583
	Han *vs*. Zhuang	0.215	0.202	0.552	0.276	0.724
	Kazakh *vs*. Mongol	0.243	0.368	0.046	0.977	0.023
	Kazakh *vs*. Tibetan	0.243	0.151	0.106	0.053	0.947
	Kazakh *vs*. Uyghur	0.243	0.175	0.534	0.267	0.737
	Kazakh *vs*. Zhuang	0.243	0.202	0.325	0.162	0.838
	Mongol *vs*. Tibetan	0.368	0.151	0.000	0.000	1.000
	Mongol *vs*. Uyghur	0.368	0.175	0.003	0.002	0.999
	Mongol *vs*. Zhuang	0.368	0.202	0.000	0.000	1.000
	Tibetan *vs*. Uyghur	0.151	0.175	0.308	0.846	0.154
	Tibetan *vs*. Zhuang	0.151	0.202	0.551	0.725	0.275
	Uyghur *vs*. Zhuang	0.175	0.202	0.607	0.304	0.697
	Mean (Std Error) | or Percentage (%) with Significant Difference	0.257 (0.014)	0.214 (0.015)	52.4%(11/21)	38.1%(8/21)	14.3%(3/21)
	**Urban lifestyles**					
	Bai *vs*. Han	0.286	0.232	0.149	0.075	0.925
	Bai *vs*. Kazakh	0.286	0.131	0.002	0.001	0.999
	Bai *vs*. Mongol	0.286	0.195	0.054	0.027	0.973
	Bai *vs*. Tibetan	0.286	0.168	0.545	0.272	0.729
	Bai *vs*. Uyghur	0.286	0.260	0.775	0.387	0.614
	Bai *vs*. Zhuang	0.286	0.193	0.033	0.016	0.984
	Han *vs*. Kazakh	0.232	0.131	0.003	0.001	0.999
	Han *vs*. Mongol	0.232	0.195	0.283	0.141	0.859
	Han *vs*. Tibetan	0.232	0.168	0.221	0.110	0.890
	Han *vs*. Uyghur	0.232	0.260	0.624	0.688	0.312
	Han *vs*. Zhuang	0.232	0.193	0.156	0.078	0.922
	Kazakh *vs*. Mongol	0.131	0.195	0.005	0.998	0.002
	Kazakh *vs*. Tibetan	0.131	0.168	0.427	0.213	0.787
	Kazakh *vs*. Uyghur	0.131	0.260	0.032	0.984	0.016
	Kazakh *vs*. Zhuang	0.131	0.193	0.039	0.981	0.019
	Mongol *vs*. Tibetan	0.195	0.168	0.507	0.254	0.747
	Mongol *vs*. Uyghur	0.195	0.260	0.284	0.858	0.142
	Mongol *vs*. Zhuang	0.195	0.193	0.875	0.438	0.563
	Tibetan *vs*. Uyghur	0.168	0.260	0.203	0.899	0.101
	Tibetan *vs*. Zhuang	0.168	0.193	0.514	0.744	0.257
	Uyghur *vs*. Zhuang	0.260	0.193	0.267	0.134	0.867
	Mean (Std Error) | or Percentage (%) with Significant Difference	0.218 (0.013)	0.200 (0.009)	28.6%(6/21)	19.0%(4/21)	14.3%(3/21)

Table S12. The means of the *Normalized Stochastic Ratio* (*NSR*) of the Chinese gut microbiomes under design **Scheme-2B** (The pair-wise ethnicity comparisons with urban & rural lifestyles combined for each comparison), and the percentage with significant difference from Wilcoxon test for the differences between different ethnicity groups in *NSR* (*P*-value=0.05)

Group	Treatment	Ethnicity1	Ethnicity2	Wilcoxon test for the NSR		
		Normalized Stochasticity Ratio (NSR)	Normalized Stochasticity Ratio (NSR)	≠	>	<
OTU-table-Phylum	Bai *vs*. Han	0.625	0.579	0.000	0.000	1.000
	Bai *vs*. Kazakh	0.625	0.452	0.000	0.000	1.000
	Bai *vs*. Mongol	0.625	0.555	0.000	0.000	1.000
	Bai *vs*. Tibetan	0.625	0.368	0.000	0.000	1.000
	Bai *vs*. Uyghur	0.625	0.488	0.000	0.000	1.000
	Bai *vs*. Zhuang	0.625	0.525	0.000	0.000	1.000
	Han *vs*. Kazakh	0.579	0.452	0.000	0.000	1.000
	Han *vs*. Mongol	0.579	0.555	0.019	0.010	0.990
	Han *vs*. Tibetan	0.579	0.368	0.000	0.000	1.000
	Han *vs*. Uyghur	0.579	0.488	0.000	0.000	1.000
	Han *vs*. Zhuang	0.579	0.525	0.000	0.000	1.000
	Kazakh *vs*. Mongol	0.452	0.555	0.000	1.000	0.000
	Kazakh *vs*. Tibetan	0.452	0.368	0.007	0.003	0.997
	Kazakh *vs*. Uyghur	0.452	0.488	0.341	0.830	0.170
	Kazakh *vs*. Zhuang	0.452	0.525	0.018	0.991	0.009
	Mongol *vs*. Tibetan	0.555	0.368	0.000	0.000	1.000
	Mongol *vs*. Uyghur	0.555	0.488	0.014	0.007	0.993
	Mongol *vs*. Zhuang	0.555	0.525	0.024	0.012	0.988
	Tibetan *vs*. Uyghur	0.368	0.488	0.000	1.000	0.000
	Tibetan *vs*. Zhuang	0.368	0.525	0.000	1.000	0.000
	Uyghur *vs*. Zhuang	0.488	0.525	0.244	0.878	0.122
Mean (Std Error) | or Percentage (%) with Significant Difference		0.540 (0.019)	0.486 (0.015)	90.5%(19/21)	71.4%(15/21)	19.0%(4/21)
OTU-table-Family	Bai *vs*. Han	0.384	0.318	0.000	0.000	1.000
	Bai *vs*. Kazakh	0.384	0.241	0.000	0.000	1.000
	Bai *vs*. Mongol	0.384	0.366	0.157	0.078	0.922
	Bai *vs*. Tibetan	0.384	0.212	0.000	0.000	1.000
	Bai *vs*. Uyghur	0.384	0.321	0.028	0.014	0.986
	Bai *vs*. Zhuang	0.384	0.284	0.000	0.000	1.000
	Han *vs*. Kazakh	0.318	0.241	0.007	0.004	0.996
	Han *vs*. Mongol	0.318	0.366	0.001	0.999	0.001
	Han *vs*. Tibetan	0.318	0.212	0.000	0.000	1.000
	Han *vs*. Uyghur	0.318	0.321	0.941	0.530	0.470
	Han *vs*. Zhuang	0.318	0.284	0.019	0.009	0.991

	Kazakh *vs*. Mongol	0.241	0.366	0.000	1.000	0.000
	Kazakh *vs*. Tibetan	0.241	0.212	0.234	0.117	0.883
	Kazakh *vs*. Uyghur	0.241	0.321	0.032	0.984	0.016
	Kazakh *vs*. Zhuang	0.241	0.284	0.169	0.916	0.084
	Mongol *vs*. Tibetan	0.366	0.212	0.000	0.000	1.000
	Mongol *vs*. Uyghur	0.366	0.321	0.156	0.078	0.922
	Mongol *vs*. Zhuang	0.366	0.284	0.000	0.000	1.000
	Tibetan *vs*. Uyghur	0.212	0.321	0.000	1.000	0.000
	Tibetan *vs*. Zhuang	0.212	0.284	0.000	1.000	0.000
	Uyghur *vs*. Zhuang	0.321	0.284	0.209	0.104	0.896
Mean (Std Error) | or Percentage (%) with Significant Difference		0.319 (0.014)	0.288 (0.011)	71.4%(15/21)	47.6%(10/21)	23.8%(5/21)
OTU-table-Genus	Bai *vs*. Han	0.322	0.266	0.000	0.000	1.000
	Bai *vs*. Kazakh	0.322	0.207	0.000	0.000	1.000
	Bai *vs*. Mongol	0.322	0.324	0.773	0.386	0.614
	Bai *vs*. Tibetan	0.322	0.193	0.000	0.000	1.000
	Bai *vs*. Uyghur	0.322	0.288	0.190	0.095	0.905
	Bai *vs*. Zhuang	0.322	0.239	0.000	0.000	1.000
	Han *vs*. Kazakh	0.266	0.207	0.042	0.021	0.979
	Han *vs*. Mongol	0.266	0.324	0.000	1.000	0.000
	Han *vs*. Tibetan	0.266	0.193	0.000	0.000	1.000
	Han *vs*. Uyghur	0.266	0.288	0.419	0.791	0.209
	Han *vs*. Zhuang	0.266	0.239	0.061	0.031	0.969
	Kazakh *vs*. Mongol	0.207	0.324	0.000	1.000	0.000
	Kazakh *vs*. Tibetan	0.207	0.193	0.479	0.240	0.761
	Kazakh *vs*. Uyghur	0.207	0.288	0.027	0.987	0.013
	Kazakh *vs*. Zhuang	0.207	0.239	0.340	0.830	0.170
	Mongol *vs*. Tibetan	0.324	0.193	0.000	0.000	1.000
	Mongol *vs*. Uyghur	0.324	0.288	0.245	0.122	0.878
	Mongol *vs*. Zhuang	0.324	0.239	0.000	0.000	1.000
	Tibetan *vs*. Uyghur	0.193	0.288	0.001	1.000	0.000
	Tibetan *vs*. Zhuang	0.193	0.239	0.013	0.993	0.007
	Uyghur *vs*. Zhuang	0.288	0.239	0.084	0.042	0.958
Mean (Std Error) | or Percentage (%) with Significant Difference		0.273 (0.011)	0.252 (0.010)	61.9%(13/21)	47.6%(10/21)	23.8%(5/21)
OTU-table-Species	Bai *vs*. Han	0.296	0.224	0.000	0.000	1.000
	Bai *vs*. Kazakh	0.296	0.176	0.000	0.000	1.000
	Bai *vs*. Mongol	0.296	0.274	0.021	0.010	0.990
	Bai *vs*. Tibetan	0.296	0.157	0.000	0.000	1.000
	Bai *vs*. Uyghur	0.296	0.238	0.013	0.007	0.993
	Bai *vs*. Zhuang	0.296	0.198	0.000	0.000	1.000
	Han *vs*. Kazakh	0.224	0.176	0.096	0.048	0.952

	Han *vs*. Mongol	0.224	0.274	0.000	1.000	0.000
	Han *vs*. Tibetan	0.224	0.157	0.000	0.000	1.000
	Han *vs*. Uyghur	0.224	0.238	0.498	0.751	0.249
	Han *vs*. Zhuang	0.224	0.198	0.052	0.026	0.974
	Kazakh *vs*. Mongol	0.176	0.274	0.000	1.000	0.000
	Kazakh *vs*. Tibetan	0.176	0.157	0.114	0.057	0.943
	Kazakh *vs*. Uyghur	0.176	0.238	0.068	0.966	0.034
	Kazakh *vs*. Zhuang	0.176	0.198	0.523	0.739	0.262
	Mongol *vs*. Tibetan	0.274	0.157	0.000	0.000	1.000
	Mongol *vs*. Uyghur	0.274	0.238	0.166	0.083	0.917
	Mongol *vs*. Zhuang	0.274	0.198	0.000	0.000	1.000
	Tibetan *vs*. Uyghur	0.157	0.238	0.001	1.000	0.000
	Tibetan *vs*. Zhuang	0.157	0.198	0.001	1.000	0.000
	Uyghur *vs*. Zhuang	0.238	0.198	0.108	0.054	0.946
Mean (Std Error) | or Percentage (%) with Significant Difference		0.237 (0.011)	0.210 (0.009)	61.9%(13/21)	52.4%(11/21)	23.8%(5/21)
